# Peer pressure from a *Proteus mirabilis* self-recognition system controls participation in cooperative swarm motility

**DOI:** 10.1371/journal.ppat.1007885

**Published:** 2019-07-19

**Authors:** Murray J. Tipping, Karine A. Gibbs

**Affiliations:** Department of Molecular and Cellular Biology, Harvard University, Cambridge, Massachusetts, United States of America; University of Washington, UNITED STATES

## Abstract

Colonies of the opportunistic pathogen *Proteus mirabilis* can distinguish self from non-self: in swarming colonies of two different strains, one strain excludes the other from the expanding colony edge. Predominant models characterize bacterial kin discrimination as immediate antagonism towards non-kin cells, typically through delivery of toxin effector molecules from one cell into its neighbor. Upon effector delivery, receiving cells must either neutralize it by presenting a cognate anti-toxin as would a clonal sibling, or suffer cell death or irreversible growth inhibition as would a non-kin cell. Here we expand this paradigm to explain the non-lethal Ids self-recognition system, which stops access to a social behavior in *P*. *mirabilis* by selectively and transiently inducing non-self cells into a growth-arrested lifestyle incompatible with cooperative swarming. This state is characterized by reduced expression of genes associated with protein synthesis, virulence, and motility, and also causes non-self cells to tolerate previously lethal concentrations of antibiotics. We show that temporary activation of the stringent response is necessary for entry into this state, ultimately resulting in the iterative exclusion of non-self cells as a swarm colony migrates outwards. These data clarify the intricate connection between non-lethal recognition and the lifecycle of *P*. *mirabilis* swarm colonies.

## Author summary

A resident of animal intestines, *Proteus mirabilis* is a major cause of catheter-associated urinary tract infections and can cause recurrent, persistent infections. Swarming, which is a collective behavior that promotes centimeter-scale population migration, is implicated in colonization of bladders and kidneys. A regulatory factor of swarming is kin recognition, which involves the transfer of a self-identity protein from one cell into a physically adjacent neighboring cell. However, *how* kin recognition regulates swarming was previously unclear. We have now shown a mechanism linking kin recognition, swarm migration, and antibiotics tolerance: cells induce a transient antibiotics-tolerant, persister-like state in adjacent non-identical cells which in turn prevents non-identical cells from continuing to participate in collective swarming. These affected non-identical cells continue to exhibit large-scale gene expression suggesting an active shift into a different expression state. These data provide two key insights for the field. First, kin recognition can be a regulatory mechanism that acts with spatial and temporal precision. Second, induction into an antibiotics-tolerant state, instead of occurring stochastically, can be physically and spatially regulated by neighboring cells. These insights highlight the importance of further developing four-dimensional (time and X-, Y-, Z-axes) model systems for interrogating cell-cell signaling and control in microbial populations.

## Introduction

Organisms rarely live in complete isolation. Living in a community can provide benefits to each individual. However, there is a constant balance between the interests of individuals and the maintenance of community-wide advantages. A stable evolutionary strategy is for individuals to preferentially direct advantages to close kin [1–3]. This behavior, known as kin discrimination, has been the subject of focused study.

Several examples of kin discrimination in bacteria have been elegantly described, including those mediated by Type IV [4], Type VI [5, 6], and Type VII [7] secretion system based effector exchange, contact-dependent inhibition (CDI) [8, 9], and the MafB toxins of the *Neisseria* [10]. One common thread between these systems is that they characterize discrimination as immediate antagonism towards cells or strains that are non-kin, typically through delivery of lethal toxin effector molecules. Upon effector delivery, receiving cells must either neutralize it by presenting a cognate anti-toxin or suffer immediate negative consequences, typically cell death [5] or permanent inhibition of growth [8]. Here we describe an expansion of these mechanisms: the Ids self-recognition system mediates kin discrimination in *Proteus mirabilis* by selectively inducing non-self cells into a growth-arrested lifestyle incompatible with social behavior, thereby controlling access to that behavior.

*P*. *mirabilis*, a major cause of recurrent complicated urinary tract infections [11], engages in several sophisticated social behaviors such as swarming on rigid surfaces. Swarms are formed by many elongated (~ 10–80 μm) “swarmer” cells moving cooperatively, allowing for colony expansion over centimeter-scale distances. Rounds of swarming are interspersed with periods of non-expansion termed “consolidation”. The oscillation between swarming and consolidation leads to a characteristic pattern of concentric rings on higher percentage agar plates [12]. Effective *P*. *mirabilis* swarming relies on the ability of swarmer cells to form large rafts that together move much more quickly than isolated individuals [13]. Rafts are fluid, transient collectives that cells frequently enter and exit. As such, an individual cell interacts with many different neighbors through the lifetime of a swarm. During swarming, *P*. *mirabilis* cells can communicate with each other by exchanging proteins through contact-dependent secretion systems [14, 15]. These signals in turn cause emergent changes in swarm behavior [16, 17].

*P*. *mirabilis* swarms exhibit the ability to recognize self in several ways. The oldest known example is Dienes line formation: two swarms of the same strain merge into a single swarm upon meeting, while two swarms of different strains instead form a human-visible boundary [18, 19]. More recently, the phenomenon of territorial exclusion was described: in a mixed swarm comprising two different strains, one strain is prevented from swarming outwards by the other [15]. Clonal swarms of *P*. *mirabilis* have a coherent self identity, minimally mediated by the Ids system encoded by six genes *idsA-F*. Deletion of the *ids* locus results in the mutant strain no longer recognizing its wild-type parent as self [20]. Several of the molecular mechanisms governing Ids-mediated self recognition have been described in detail. However, how the Ids system functions in local behaviors has remained elusive.

**Fig 1:**
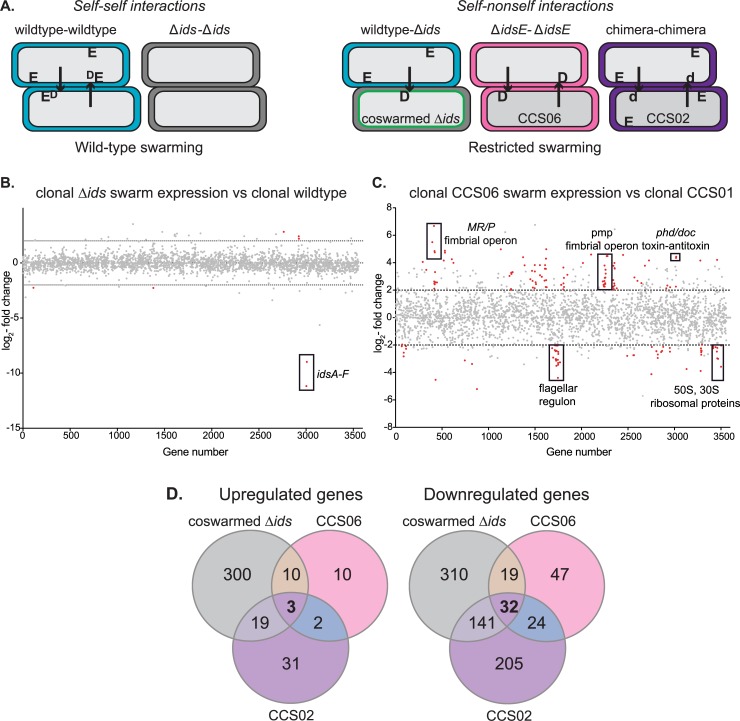
Ids mismatch during swarming causes large transcriptional shifts. A. Cartoons show self versus non-self interactions as defined by the Ids state in and between cells. The post-transferred state of IdsD is depicted. Endogenous IdsD and IdsE are represented by “D” and “E”, while a non-self variant of IdsD is represented by “d.” B. A graphical representation of genes differentially regulated in consolidator cells isolated from clonal Δ*ids* swarms versus consolidator cells from clonal wild-type swarms. The X-axis corresponds to each gene on the *P*. *mirabilis* BB2000 chromosome. The Y-axis corresponds to log_2_-fold difference in relative transcript abundance. Genes with significantly different relative transcript abundance, defined as fold-change > log_2_ 1.5, p < 0.05, are marked in red. All data are included. The genes *idsA-F*, which were deleted to construct the Δ*ids* strain, are noted. C. A graphical representation of genes differentially regulated in whole swarms of strain CCS06 versus consolidators from strain CCS01, constructed similarly to that of (B). CCS01 is the wild-type equivalent background for CCS06 and CCS02 as the *ids* genes are expressed from a plasmid in all strains; the strain background is Δ*ids*. Labeled genes are discussed in the text. D. Venn diagrams showing genes similarly differentially regulated in three separate swarm conditions for an Ids mismatch between cells: co-swarmed Δ*ids*, clonal CCS06, and clonal CCS02. Two biological repeats were performed.

Briefly, two proteins, IdsD and IdsE, govern self identity. IdsD is transferred between cells in a Type VI secretion system (T6SS)-dependent fashion; disruption of the T6SS prevents all Ids signal transfer [17, 21]. A cell in a swarm is considered to be self if it produces an IdsE protein that can bind IdsD proteins sent from neighboring cells. Disruption of these IdsD-IdsE interactions, either through deletion of *idsE* or through production of non-self IdsD or IdsE variants, result in strains that display extreme attenuation in swarm expansion without loss of viability [16, 17]. Fig 1A shows cartoon representations of Ids-mediated self recognition, where endogenous IdsD and IdsE are represented by capital letters “D” and “E”, and a non-self variant of IdsD is represented by lowercase “d.” The IdsD protein must be incoming: endogenous IdsD and IdsE proteins produced within a single cell do not impact self-recognition behaviors [21]. We describe conditions that lead to non-self recognition as “Ids mismatch.”

Crucially, territorial exclusion by Ids does not affect viability. Excluded cells grow and divide at a comparable rate to non-excluded cells when isolated from swarms [17]. While Ids has been described as a toxin-antitoxin system [22, 23], this characterization is inconsistent with experimental data and is likely due to the reliance on the T6SS for transport of IdsD [17, 21]. The T6SS is often characterized as a lethal toxin delivery mechanism [24]. A mechanistic description of Ids-mediated recognition is needed to reconcile the data and would provide a model for other non-lethal mechanisms that might be attuned for surface-dwelling swarm migration.

Here we show that even though an Ids recognition signal transfer happens while cells are actively migrating as a swarm, the recognition response is delayed until cells have stopped moving. We show that recognition of non-self is at least partially mediated by ppGpp levels within the cell, and this contributes to a concerted shift of cells into a distinctive, antibiotic-tolerant state that is incompatible with continuation of swarming. We found that this Ids-induced state is short-acting; induction requires continuous cell-cell interactions. In the context of a swarm, the collective consequence is an iterative winnowing of the non-self cells from the swarm fronts during periods of no active migration. We posit that the cell-cell communication of these non-lethal factors therefore acts as a control system during swarm expansion by diverting non-self from developing into swarm-compatible cells and thus preventing non-self cells from taking part in cooperative swarm behavior.

## Results

### Ids mismatch during swarming induces a distinct transcriptional state

We initially sought to determine the method through which Ids caused non-self cells to be territorially excluded from swarms. Given the lack of lethality and the stark attenuation of swarm colony expansion observed during Ids-mediated territorial exclusion [15, 17], we hypothesized that an Ids mismatch caused broad changes in gene expression of the recipient cell. Ids mismatch is defined here as transcellular communication of IdsD to a recipient cell lacking a cognate IdsE. Therefore, we performed RNA-Seq differential expression analysis using conditions that would produce either self or non-self interactions, all within genetically equivalent backgrounds. We isolated total RNA from cells undergoing consolidation, because consolidation is the swarm development stage most tightly connected to major transcriptional changes [25].

As a baseline, we compared transcriptional profiles between cells from clonal swarms of wildtype and independently, of a derived mutant strain lacking the *ids* genes (BB2000::*ids*Ω*Cm* [20], herein referred to as “Δ*ids*”). The swarm colonies of both strains expand equivalently and have no notable morphological differences [20]. A complete list of genes with differential transcript abundance is found in S1 Table. Overall, few differences were apparent between clonal swarms of wildtype and the Δ*ids* strain. 10 genes were found to be significant (fold-change > log_2_ 1.5, p < 0.05), six of which were the *ids* genes deleted in the construction of the Δ*ids* strain (Fig 1B).

We next considered differences in strains experiencing Ids mismatch. We examined three different conditions: a clonal swarm in which every cell lacked the *idsE* gene (CCS06), a clonal swarm in which every cell is a chimera containing an IdsE protein unable to bind transferred IdsD proteins (CCS02), and cells of a Δ*ids*-derived strain constitutively producing Green Fluorescent Protein GFPmut2 (Δ*ids*-GFP) that were isolated from a 1:1 co-swarm with wildtype through fluorescent-activated cell sorting (FACS), henceforth termed “co-swarmed Δ*ids*". Strains CCS06 and CCS02 are the Δ*ids* background containing a plasmid expressing the *ids* operon under its native promoter; mutations to the *ids* genes were made in the plasmid-based allele. All strains have previously been verified and characterized [16, 17, 20, 21]. As the two clonal swarm colonies have attenuated expansion, we were only able to harvest whole colonies as visible consolidation phases were less distinct. RNA-Seq differential expression analysis was performed on cells from each condition as compared to appropriate control samples: co-swarmed Δ*ids* were compared with clonal Δ*ids*, and CCS02 and CCS06 swarms were compared to a clonal Δ*ids*-derived swarm in which cells expressed plasmid-encoded *ids* genes (CCS01, Δ*ids* pids_BB_). Large-scale changes to relative transcript abundances were apparent for each condition: 231 genes in CCS06, 457 genes in CCS02, and 836 genes in co-swarmed Δ*ids*, which represents approximately 6%, 13%, and 23% of total genes, respectively (Fig 1, S2 Table, S3 Table, S4 Table).

General trends were apparent even though differences in relative abundance of transcripts were present in a diverse and widespread range of genes. We observed a concerted decrease in transcripts for class I, II, and III genes for flagellar synthesis such as *flhDC*, *filA*, and *fliC*. We also observed a decrease in transcripts for many genes associated with protein synthesis, such as the 50S ribosomal protein *rplT* and 30S ribosomal protein *rpsP*, along with ribosomal-associated elongation factors such as EF-Tu. Several genes involved in respiration, including those of the F_o_F_1_ ATP synthase, also had significantly fewer transcripts as did those transcripts for different virulence-associated protein families [26–31], such as *hpmA*, *umoA*, and *zapD*. Overall, fewer genes had an increased relative abundance as compared to the control strains: 109 genes in CCS06, 56 genes in CCS02, and 332 genes in co-swarmed Δ*ids*, respectively. Within these genes, several endogenous toxin-antitoxin systems displayed an increased relative abundances of transcripts, including genes that encode homologous proteins to *Escherichia coli* YfiA (also known as RaiA) [32] and to toxin/antitoxin pairs ParE/CC2985 [33] and Phd/Doc [34]. There was also an increased relative abundance for several fimbriae families, including genes encoding MR/P and P-like fimbria. A large proportion of differentially regulated genes were proteins of unknown function.

A subset of differentially abundant genes was shared among all three datasets; these were representative of families found in each Ids mismatch strain. Three genes had increased relative abundance in transcripts as compared to wildtype; 32 genes had decreased (Fig 1D). The 32 genes with decreased relative transcripts included those involved in motility, chemotaxis, ribosomal proteins, and metabolism (S5 Table). Of the three genes with increased relative transcripts, two encode the Phd/Doc endogenous toxin-antitoxin system. The third gene is *rob*, which has been associated with the induction of low-metabolism states in *E*. *coli* [35]. Thus, cells under the influence of incoming IdsD, and without a cognate IdsE protein present, enter a distinctive transcriptional state from either wild-type or Δ*ids* cells participating in a normal swarm cycle. Transcriptional shifts of these types are often associated with entry into altered states induced by a variety of environmental and temporal cues, including nutrient and membrane stress [36].

### Cells experiencing Ids mismatch display increased tolerance to lethal concentrations of antibiotics

We hypothesized that the changes in Ids-excluded cells might also result in the secondary effect of increased antibiotic tolerance. We used susceptibility to antibiotics as a proxy for whether cells have entered an altered state. We conducted antibiotic tolerance assays on wildtype, the Δ*ids* strain, and a third strain containing an unmarked in-frame (nonpolar) deletion of the chromosome-encoded *idsE* within the wildtype background [21]. The Δ*ids* strain encodes a transgenic resistance gene to chloramphenicol; the deletion is stable in growth media without chloramphenicol. Neither the wildtype nor the Δ*idsE* strain encode transgenic genes for antibiotics resistance. All three strains are endogenously resistant to tetracycline.

**Fig 2:**
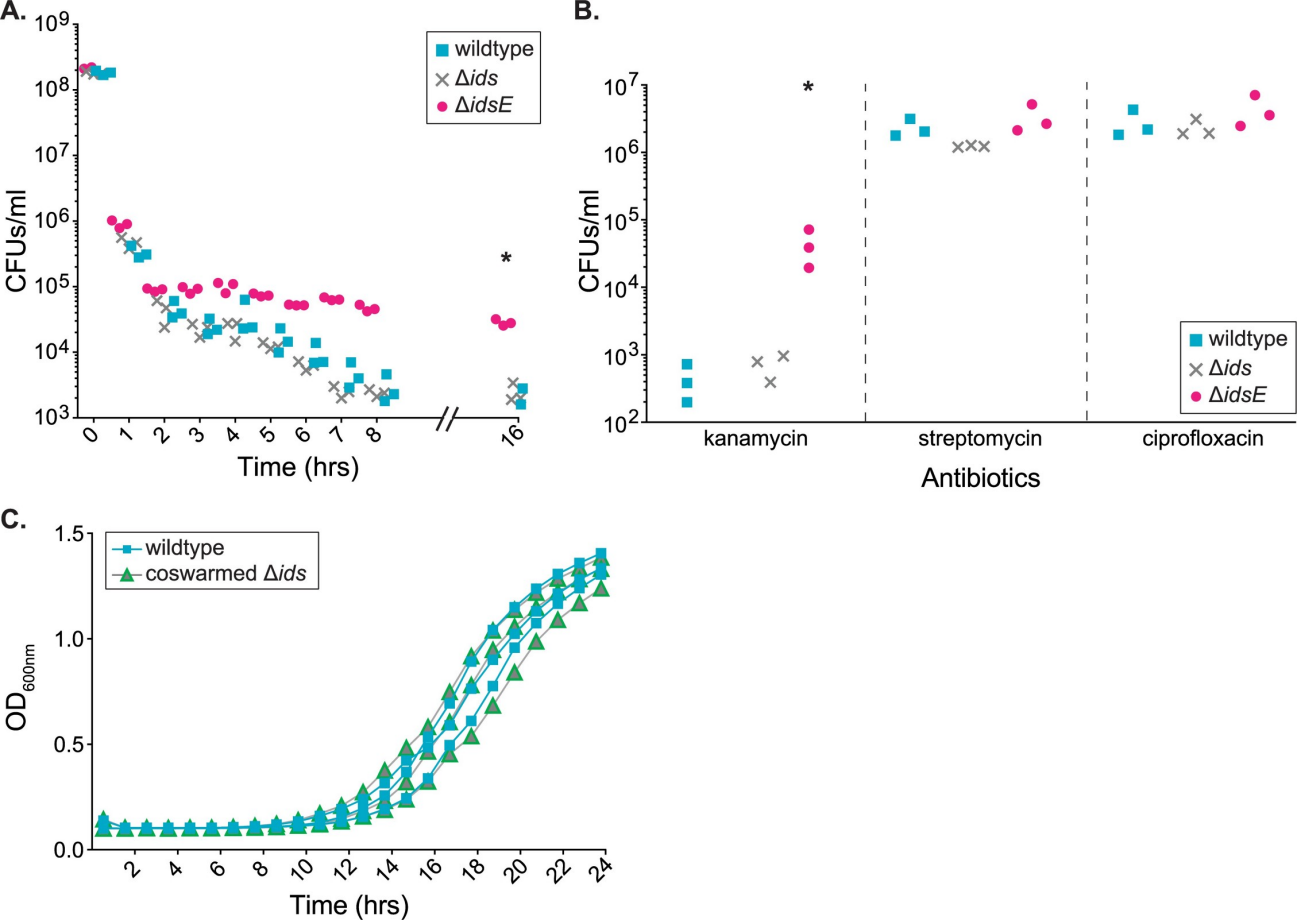
Cells experiencing Ids mismatch display transient tolerance to lethal concentrations of antibiotics. A. Killing curves for cells of wildtype (cyan square), Δ*ids* (gray "x"), and Δ*idsE* (magenta circle) harvested from swarm plates and exposed to 100 μg ml^-1^ ampicillin. Note that the Y-axis is a logarithmic scale. Asterisks represent statistically significant difference between Δ*idsE* and wildtype (p = 0.02) calculated using a Mann-Whitney test. B. Survival of strains wildtype (cyan square), Δ*ids* (gray “x”), and Δ*idsE* (magenta circle) after swarm colonies were harvested and exposed to 60 μg ml^-1^ kanamycin (left), 50 μg ml^-1^ streptomycin (middle), and 1 μg ml^-1^ ciprofloxacin (right) for 12 hours. Asterisks represent statistically significant difference between Δ*idsE* and wildtype (p = 0.04) calculated using a Mann-Whitney test. C. Territorial exclusion does not result in long-term growth defects. Optical density at 600 nm (OD_600 nm_) was measured over time for liquid cultures of wildtype and the co-swarmed Δ*ids* strain. Liquid cultures were inoculated using cells isolated by FACS from co-swarm colonies where the Δ*ids* strain had been actively excluded from the swarm front.

We predicted that swarms of the Δ*idsE* strain would display increased antibiotic tolerance compared to wildtype and the Δ*ids* strain. We first tested the beta-lactam antibiotic ampicillin to which the wild-type BB2000 is susceptible. We used independent, clonal swarms of either wildtype, the Δ*ids* strain, or the Δ*idsE* strain, each grown in the absence of antibiotics. We harvested cells after the entry to the third swarm ring. Cells were resuspended in LB media and immediately subjected to 100 μg ml^-1^ ampicillin exposure. Samples were extracted for viability assays on fresh media lacking antibiotics at regular intervals until eight hours and again at 16 hours. No clear difference was observed between wildtype and the Δ*ids* strain at any timepoint (Fig 2A). Wildtype and the Δ*ids* strain exhibited a killing of approximately 10^5^-fold after eight hours, while the Δ*idsE* strain experienced a killing of approximately 10^4^-fold (Fig 2A). Under these conditions, the Δ*idsE* strains had an approximately 50-fold increase in survival as compared to wildtype, even after sixteen hours incubation in ampicillin (Fig 2A).

We repeated this assay with three additional antibiotics to explore whether the Δ*idsE* cells exhibited a broad tolerance to antibiotics. We utilized the fluoroquinolone ciprofloxacin and the aminoglycosides streptomycin and kanamycin. No difference between wildtype, the Δ*ids* strain, and the Δ*idsE* strain was observed in viable cell counts following 50 μg ml^-1^ streptomycin or 1 μg ml^-1^ ciprofloxacin exposure after 12 hours (Fig 2B). These antibiotics resulted in lower rates of cell killing as compared to ampicillin, likely reflecting a higher native resistance of *P*. *mirabilis* to these drugs [37, 38]. However, the Δ*idsE* strain showed an increased number of viable cells as compared to wildtype or the Δ*ids* strain after 60 μg ml^-1^ kanamycin incubation for 12 hours (Fig 2B). Therefore, the Δ*idsE* strain displays increased tolerance to both kanamycin and ampicillin under these conditions, indicating limited resistance to antibiotics.

Since Ids functions through cell-cell contact-dependent secretion of the identity marker IdsD [15, 17], we next tested whether IdsD secretion was required for antibiotic tolerance to emerge. Cells secrete IdsD into growth media [15]. We performed this assay using MJT01, which is an Δ*idsE*-derived strain containing a non-functional T6SS [17, 39] and so does not secrete IdsD. The deletion of *idsE* and the disruption of the T6SS-encoding gene *tssB/vipA* are unmarked and nonpolar on the chromosome. We observed no clear difference between MJT01 and wildtype over three biological repeats (S1 Fig). To examine whether social exchange was causative for antibiotics tolerance, we tested cells of the Δ*idsE* strain that were grown to stationary phase with shaking in liquid. IdsD transfer between cells is limited, if at all, during liquid growth. We observed no difference in antibiotics tolerance for the Δ*idsE* strains as compared to wildtype over three biological repeats (S2 Fig). Therefore, antibiotics tolerance was induced by an Ids mismatch and caused by the transfer of IdsD between neighboring cells without a cognate IdsE present.

We considered whether this Ids-mediated antibiotic tolerance might be due to entry into an irreversible state. To examine the dynamics of how cells exit from an Ids mismatch, we assayed co-swarms in which the GFP-producing Δ*ids* strain (Δ*ids*-GFP) was inoculated with an equal amount of a wild-type strain constitutively producing DsRed (wildtype-DsRed). We let the swarm progress to the third swarm ring and then harvested the swarms. Cells of each strain were immediately sorted with FACS. Analysis of sorted cells showed that Δ*ids-*GFP formed ~10% of the sample from the third swarm ring. Equal numbers of particles of each strain were inoculated in LB media without antibiotics, and growth at 37°C was measured for 24 hours through optical density at 600 nm. No differences between co-swarmed wildtype-DsRed and co-swarmed Δ*ids*-GFP were observed at any time-point (Fig 2C). We concluded that the effects induced by an Ids mismatch are transient outside of continual contact-mediated pressure. Consistent with this assertion, we found no differences between the growth of CCS02 (the chimera Ids mismatch strain) and strain CCS01 grown in liquid LB media at 37°C (S3 Fig). IdsD transfer does not occur, or is limited, during liquid growth. Therefore, an individual cell within a swarm is shifted into a distinct transcriptional state when it has received non-self IdsD from the surrounding cells. We found that this cell state caused by an Ids mismatch is temporary and reversible.

### Ids recognition mechanisms induce and require the stringent response

**Fig 3:**
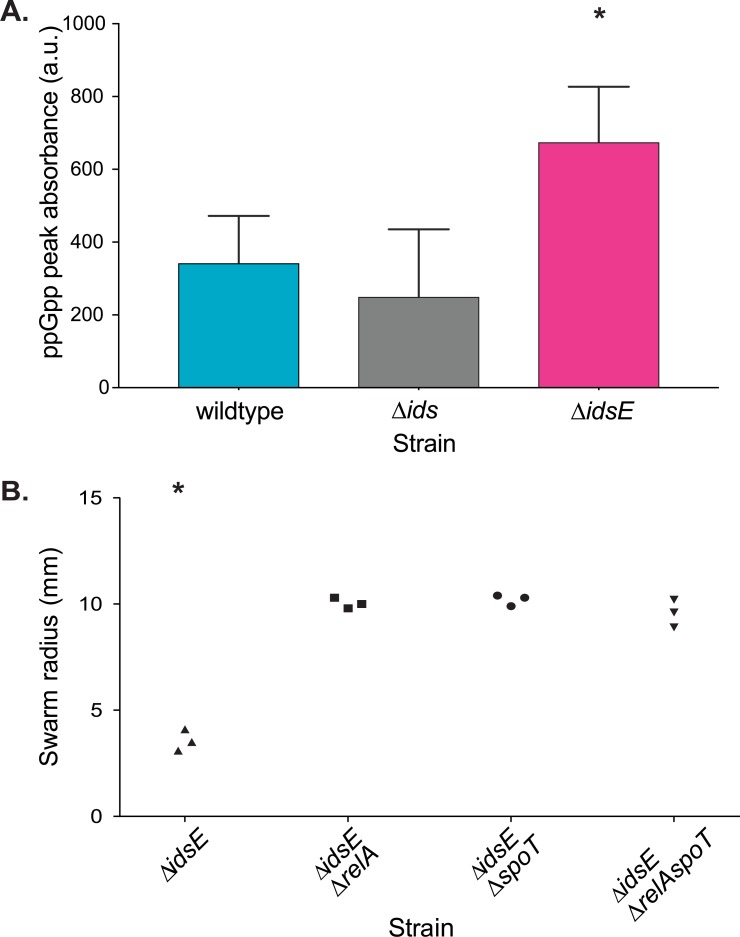
Cells affected by Ids mismatch induce an increase in ppGpp; the *relA* and *spoT* genes are necessary to induce Ids-mediated territorial exclusion. A. Mean ppGpp absorbances from wildtype, the Δ*ids* strain, and the Δ*idsE* strain, calculated by integrating areas under peaks. Errors show standard deviations. Asterisks show statistically significant difference (p = 0.03) between Δ*idsE* and wildtype, calculated through a Mann-Whitney test. B. Swarm colony radius expansion of the Δ*idsE*, Δ*idsE*Δ*relA*, Δ*idsE*Δ*spoT*, and Δ*idsE*Δ*relA*Δ*spoT* strains after 16 hours of swarming. All replicates are shown. Asterisks show statistically significant difference between strains (p = 0.02) calculated through a Kruskal-Wallis test.

Entry into an antibiotic-tolerant state has been linked in other bacteria to the stringent response [36, 40], mediated by the alarmone messenger molecule (p)ppGpp. Although the stringent response has not been studied in *P*. *mirabilis*, the genome for wild-type BB2000 contains two canonical genes for production and degradation of ppGpp, *relA* and *spoT* [41]. We tested whether Ids mismatch was connected to the stringent response. Using quantitative high performance liquid chromatography (HPLC) [42], we directly measured total ppGpp quantities in cells independently harvested from swarms of clonal wildtype, clonal Δ*ids*, or clonal Δ*idsE*. Nucleotide samples were purified, separated by HPLC, and quantified by measuring UV absorbance spectra using established methods [42]. We performed three biological repeats of ppGpp measurements and found that the samples from the Δ*idsE* strain contained nearly twice the ppGpp levels as wildtype and the Δ*ids* strain (Fig 3A, S4 Fig). These results indicate that ppGpp levels and Ids mismatch are linked.

We next examined whether ppGpp is necessary for Ids mismatch, specifically focusing on the Δ*idsE* swarm deficiency. Deletions of *relA* or *spoT* can prevent ppGpp accumulation and as such, prevent cells from activating the stringent response in several bacteria as discussed in [43]. We constructed three Δ*idsE*-derived strains, each with an independent, unmarked, nonpolar chromosomal deletion of *relA*, *spoT*, or both. We quantified ppGpp levels in each strain using HPLC as described above and found that ppGpp levels in each strain were minimal as compared to wildtype (S5A Fig). We next assayed each strain for swarm colony expansion as compared to that of wildtype and the parent Δ*idsE* strain. We found that each newly constructed strain formed swarms of a diameter equivalent to wildtype and nearly twice that of the parent Δ*idsE* strain (Fig 3C). We also found that in 1:1 co-swarms with the wild-type strain, the ppGpp-deficient strains were excluded from the swarm colony edges, similar to the Δ*idsE* strain (S5B Fig). We interpret these results as interactions with the wild-type strain are not equivalent to interactions with clonal cells lacking ppGpp. Therefore, while ppGpp is required for Ids mismatch to have an effect in clonal swarms, it is likely not the only factor. Moreover, we posit that ppGpp might be a causative factor upstream of the observed transcriptional and physiological changes.

### The transcriptional shift observed in cells experiencing Ids mismatch occurs during non-swarming consolidation periods

Having observed that a response to Ids mismatch is only present under consistent pressure from neighboring non-self cells (Fig 2), we reasoned then that the effects of Ids mismatch control might be spatially and/or temporally attuned. The small molecule ppGpp might allow for such a rapid and transient response in cells. To interrogate this model, we took advantage of the genes newly identified as being induced in the presence of non-self (Fig 1) to develop a fluorescent transcriptional reporter system. A gene encoding a variant of the fluorescent protein Venus [44] was engineered to be inserted immediately downstream of the gene *BB2000_0531*, resulting in Venus production being controlled by the upstream promoter. The *BB2000_0531* gene, encoding a putative sigma-54 modulation protein, was chosen as it displayed increased expression under different Ids mismatch conditions (Fig 1B, S2 Table, S3 Table, S4 Table). The reporter construct was inserted unmarked and nonpolar in the chromosome of the Δ*ids* strain, resulting in strain MJT02; this strain had no apparent growth or swarm defects.

**Fig 4:**
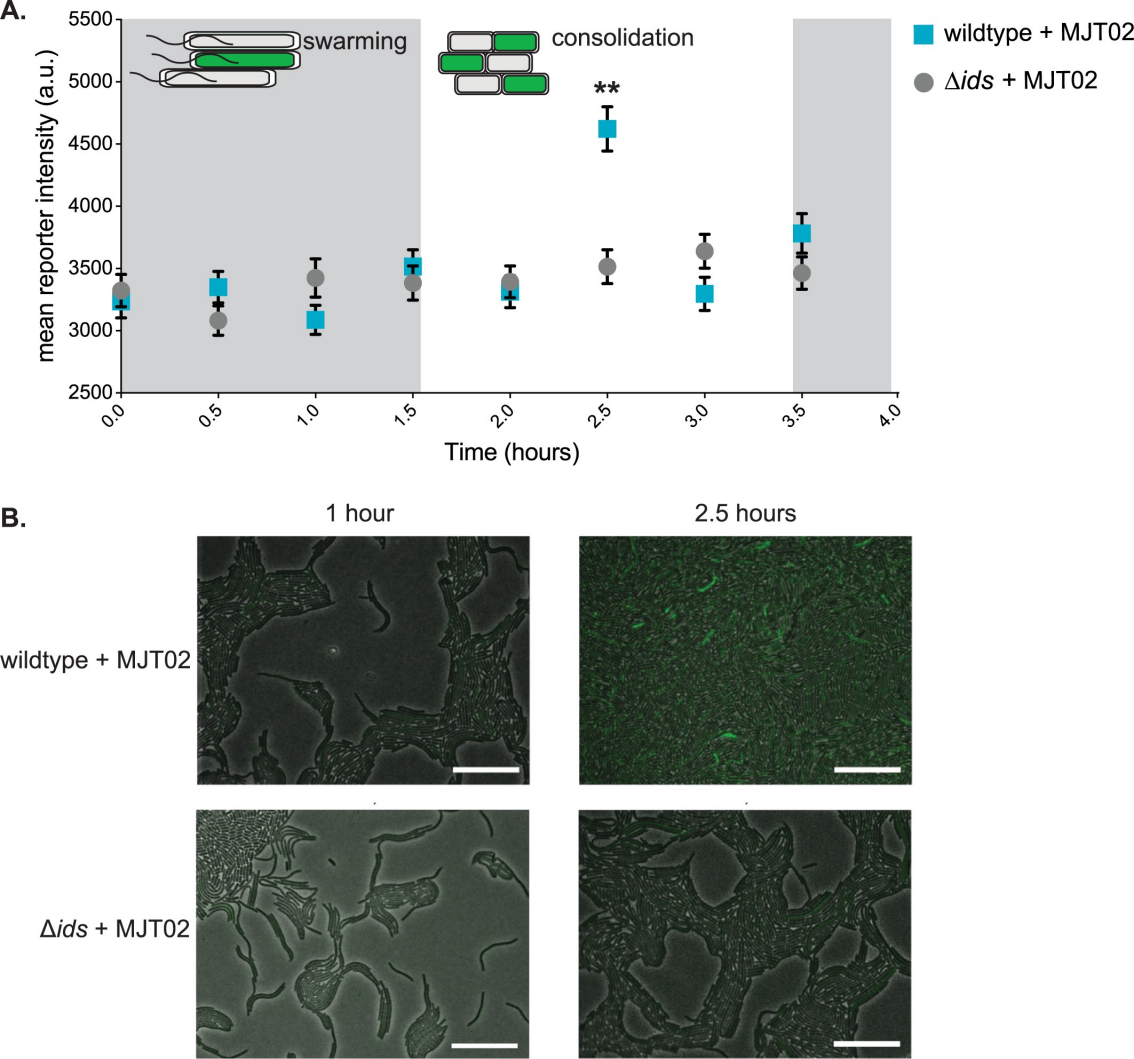
The transcriptional shift observed in cells experiencing Ids mismatch occurs during consolidation periods between swarming. A. A time-course graph showing mean swarm fluorescence intensity over time for two conditions: Δ*ids* carrying a chromosomal *BB2000_0531* fluorescence reporter (MJT02) co-swarmed with wildtype (wildtype + MJT02, cyan squares) or co-swarmed with the Δ*ids* strain (Δ*ids* + MJT02, gray circles). Three biological repeats were performed. Fluorescence was measured over equivalent areas for each experimental condition. Error bars are standard deviations; a.u. means arbitrary units. Asterisks show statistically significant differences (p < 0.01) calculated through individual Mann-Whitney tests for each timepoint. B. Representative microscopy images phase contrast overlaid with Venus fluorescence (100x magnification) of wildtype + MJT02 (top) and Δ*ids* + MJT02 (bottom) co-swarms. Images shown are of the first active swarm phase (left, 1 hour) and the first period of consolidation (right, 2.5 hours). Scale bars = 10 μm.

We performed fluorescence microscopy time-course experiments on mixed swarms to measure transcriptional changes associated with *BB2000_0531* over the course of a swarm-consolidation cycle. Two co-swarm conditions were used. In the first, a mixed culture of 50% MJT02 and 50% the Δ*ids* strain was used to inoculate swarm-permissive agar; in the second, a mixed culture of 50% MJT02 and 50% wildtype-DsRed was used. Swarms were grown at 37°C until the first swarm expansion was visible. Venus fluorescence intensity was measured at 30-minute intervals thereafter in swarm areas, and the mean fluorescence was calculated. The fluorescence intensity for both co-swarm conditions was graphed (Fig 4A); representative images are in Fig 4B. We observed a temporal spike in fluorescence associated with *BB2000_0531* correlated with the consolidation cycle; this increase was only apparent when Δ*ids*-derived cells were intermingled with wild-type cells (Fig 4). Therefore, the gene expression response to Ids mismatch occurs during consolidation.

In addition to causing territorial exclusion in mixed swarms, Ids mismatch determines boundary formation after collision between two clonal swarms [20]. Boundary formation following swarm contact is a complex process likely involving the contribution of several lethal and non-lethal systems [14, 15, 20, 45]. To test whether equivalent transcriptional shifts were observed during the initial stages of boundary formation, when cells of each strain are first in contact with one another, we measured fluorescence intensity associated with *BB2000_0531* in MJT02 swarms following collision with wildtype and Δ*ids* swarms. We observed a mean increase in fluorescence intensity over several hours after MJT02 encountered wildtype, but not in encounters with the Δ*ids* strain (S6 Fig). The observed increase in fluorescence intensity occurred before a boundary was visually apparent. In fact, formation of a visible boundary between the two strains did not occur for a further 12–18 hours after the end of this experiment, which is consistent with previous observations [18]. Therefore, the Ids mismatch induces a response in the initial stages of self versus non-self recognition, after non-self cells have interacted. We reasoned that Ids mismatch control might be relevant for the formation and/or development of a swarm colony, which is consistent with our prior hypothesis that Ids impacts cooperative behavior [17].

### Non-self cells are iteratively winnowed from the swarm front

**Fig 5:**
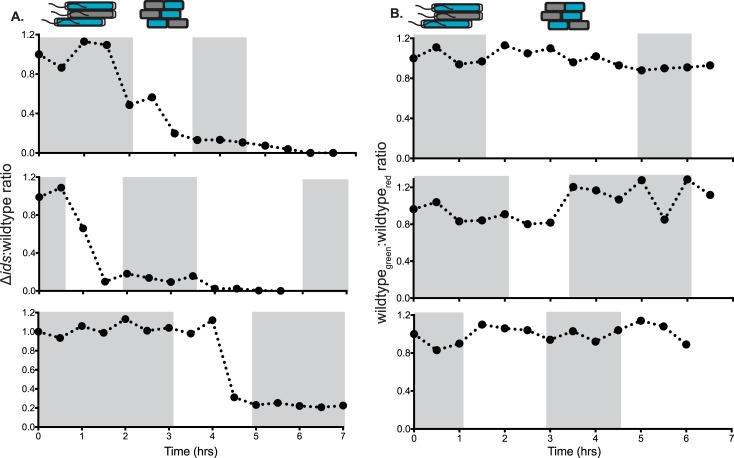
The Δ*ids* strain is excluded during consolidation between periods of swarming. (A) 1:1 co-swarms of Δ*ids*-GFP and wildtype-DsRed or (B) wildtype-GFP and wildtype-DsRed were analyzed for GFP-associated and DsRed-associated fluorescence over the course of outward migration and through progression of the swarm-consolidation cycle. Each data point indicates the proportion of GFP-producing cells measured at the swarm front at a given time after swarm emergence. Periods of active swarm expansion were determined by eye and are highlighted in gray. Three biological repeats were performed, and each is shown.

Swarming is fundamentally a collective behavior. The spatial expansion of a wild-type swarm is connected to the oscillatory developmental cycle of outward migration and non-motile consolidation. To assess the hypothesis that the Ids system likely impacts local cell-cell interactions at the boundary and within an expanding swarm, we examined territorial exclusion *in situ* using epifluorescence microscopy and utilized co-swarms constructed with equal ratios of the Δ*ids* and wild-type strains. To allow visualisation of individual cells, 10% of Δ*ids* and wildtype cells in the starting mixture constitutively expressed GFP and DsRed fluorescent proteins, specifically strains Δ*ids*-GFP and wildtype-DsRed, respectively (Fig 5). The control experiment consisted of a co-swarm in which a starting inoculum consisted of wildtype doped with 10% wildtype constitutively producing GFPmut2 (strain wildtype-GFP) and 10% wildtype-DsRed (Fig 5B). Once swarmer cells emerged from the inoculum, the proportion of cells expressing each fluorophore was measured at half-hour intervals (Fig 5). The developmental stages of active outward motility versus no outward motility (i.e., consolidation) were noted by eye (S7 Fig).

We calculated the fluorophore ratios over time for both the Δ*ids*-GFP:wildtype-DsRed and wildtype-GFP:wildtype-DsRed co-swarms for three biological repeats. The GFP/DsRed ratios in the wildtype-GFP:wildtype-DsRed control experiment did not deviate over time, with approximately equal numbers of each strain observable in the swarm over the course of eight hours (Fig 5B). The Δ*ids*-GFP:wildtype-DsRed co-swarm did not show measurable changes until well after swarm emergence (Fig 5A), indicating that Δ*ids*-GFP cells arising from the inoculum were not excluded from swarm behavior. However, large decreases in the GFP/DsRed ratio were observed in consolidation periods between rounds of swarming, starting in the first consolidation phase (Fig 5A). Later swarm rings often contained no observable Δ*ids-*GFP cells.

To test whether the ratio changes observed over multiple swarm cycles were caused by lysis of the cells experiencing Ids mismatch, we monitored a single field of view of Δ*ids*-GFP:wildtype-DsRed or Δ*ids*-GFP:Δ*ids* co-swarms over one consolidation phase. Images were taken at 5-minute intervals from the start to end of the consolidation phase, with three biological repeats performed (S8 Fig). Cell lysis rates were less than 0.1% for both conditions, with no difference in cell lysis rates apparent at any point. We did not observe an absolute decrease in Δ*ids*-GFP cell numbers (S8A Fig). We also found that as the consolidation zone increased, the ratio of fluorescence associated with Δ*ids*-GFP decreased in co-swarms with wildtype and remained constant in co-swarms with Δ*ids* (S8B Fig). Observation of individual cells in consolidation areas suggests that the Δ*ids*-GFP strain has apparent differences in cell division dynamics based on whether in a co-swarm with wildtype (non-self) or with the Δ*ids* strain (no signal). Altogether, we found that Ids-mediated exclusion was increasingly effective over the course of the co-swarm with initial equal ratios of cells, eventually resulting in Δ*ids*-GFP cells being excluded from the leading edges of swarming colonies (Fig 5). Therefore, Ids mismatch results in cells unable to proceed through the swarm developmental cycle during the consolidation period.

As Ids-mediated exclusion was correlated with consolidation in *P*. *mirabilis* swarms, we generated “hyperswarming” wild-type and Δ*ids* strains (named “wildtype-_HS_” and “Δ*ids*-_HS_,” respectively) that continually swarm outwards without consolidation [28], which leads to rapid surface coverage through swarm colony expansion. We performed co-swarms to test whether hyperswarming protected the Δ*ids*-derived strains from exclusion by wildtype. We observed that neither Δ*ids*-_HS_:wildtype-_HS_ nor Δ*ids*-_HS_:wildtype co-swarms resulted in the exclusion of the hyperswarming Δ*ids* strain (S9 Fig). However, Δ*ids*:wildtype-_HS_ co-swarms, in which the Δ*ids* strain enters consolidation, did result in territorial exclusion of Δ*ids*-derived cells (S9 Fig). We concluded that outside of the consolidation phase, the Δ*ids*-derived cells that received non-self signals were not effectively excluded, indicating that Ids mismatch does not affect swarm performance in hyperswarming cells. We propose that Ids mismatch induces the recipient cell to experience a growth arrest in the swarmer cell developmental cycle, which then prevents cells from re-entry into swarm-compatible states.

### Conclusions

**Fig 6:**
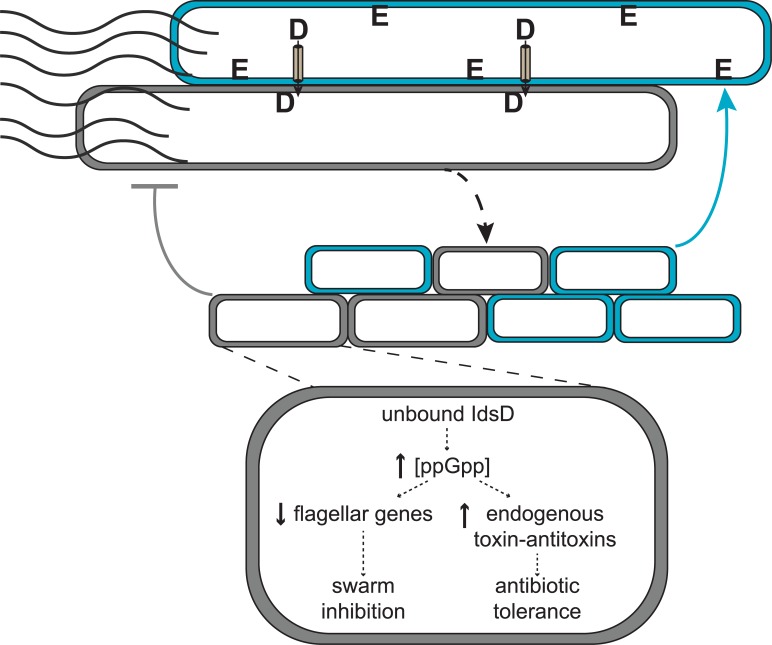
A model for Ids-mediated territorial exclusion. Top: IdsD is exchanged between motile, elongated swarmer cells. Transfer from a wildtype cell (cyan) to a Δ*ids* cell (gray) is shown. Middle, wild-type cells expressing a cognate IdsE do not experience any effects from incoming IdsD and progress normally through consolidation phase, forming new swarmer cells. The Δ*ids* cells without a cognate IdsE (gray) continue to swarm normally after IdsD transfer until entry into consolidation phase. Bottom, upon differentiation into consolidation phase, unbound IdsD induces elevated ppGpp levels and profound transcriptional changes, resulting in a growth-arrested state incompatible with differentiation into new swarmer cells. The resultant phenotype exhibits secondary effects including increased antibiotic tolerance.

Here we expand on models of kin discrimination [5, 8] by showing that the Ids system encompasses a complex and subtle recognition that is attuned to the challenges of rapid migration as a collective along a hard surface. Ids-mediated recognition controls the spatial location of non-self cells over the lifetime of a swarm. It appears that for this robustly swarming bacterium, access to a social behavior is impeded via a non-lethal mechanism: the Ids self-recognition system selectively induces non-self cells into a growth-arrested lifestyle incompatible with cooperative swarming. Intriguingly, Ids-like proteins are encoded within the genomes of other members of the *Morganellaceae* family, suggesting that this mechanism might be more broadly found. Further, these data suggest a model for Ids territorial exclusion in mixed swarms (Fig 6). IdsD is likely primarily transferred during active swarming when the secretion machinery is produced and abundantly visible [21, 25, 39]. During consolidation phase, the presence of IdsD with an absence of a cognate IdsE (resulting in unbound IdsD in recipient cells) causes a shift into a distinctive transcriptional state (Fig 1) that is partially due to activation of the stringent response via elevated ppGpp levels (Fig 3). This shifted state also causes a phenotype in affected cells that allows increased antibiotic tolerance (Fig 2). We propose that Ids mismatch functions by diverting cells, via growth arrest, from re-entry into *P*. *mirabilis’* swarm-consolidation developmental cycle, which results in individual non-self cells being iteratively winnowed out of the migrating swarm front when initially present in equal ratios (Fig 6).

Several potential models could explain exactly how unbound IdsD affects the recipient cell. One model is that the presence of unbound IdsD in a recipient cell interrupts a checkpoint in the differentiation from a swarmer to a consolidated cell or vice-versa. While the transcriptomic data provides a reasonable starting point, the list of differences for each Ids mismatch condition as compared to wildtype is quite large. These changes do not resemble those previously described during swarm-consolidation transitions [25], suggesting that Ids mismatch induces entry into a novel expression state. It is also possible that IdsD might accumulate in the membrane over time, leading to a general stress response. However, several pieces of data contradict such a model. First, non-self cells are able to escape Ids-mediated territorial exclusion under laboratory conditions by overexpression of the master flagellar regulator *flhDC*, which abolishes consolidation to form hyperswarmer cells [31]. Hyperswarming Δ*ids* cells receiving a non-self signal are motile and able to swarm with wildtype (S9 Fig). Excluded Δ*ids* cells are still able to grow and divide *in situ* [17]. Further, deletion of *relA* and/or *spoT*, which reduces ppGpp levels, allows for increased swarm colony expansion of the clonal Ids mismatch strain, Δ*idsE*, thereby bypassing Ids mismatch control (Fig 3 and S5A Fig). The molecular mechanisms for how ppGpp levels might cause attenuated expansion remain to be uncovered. Although recognition signals need flagellar regulation and internal ppGpp levels to be effective, how these pathways intersect remains to be uncovered. Interpretation of our research is further complicated, because little is published about the stringent response and/or ppGpp activity in *P*. *mirabilis*.

There are several transcriptional changes in the Ids mismatch-induced state that are not readily explained by the ppGpp response. We anticipate that ongoing studies into the emergence of bacterial dormancy and related phenotypes [46–49] in other species might help to untangle the order and hierarchy of the Ids-induced changes described here. Several candidate pathways for further analysis are apparent from the transcriptomics datasets. The role of the signaling molecule c-di-GMP in regulating motile/sessile lifestyle changes in many bacteria is well-studied and an attractive target for future work [50]. The SOS response, mediated by RecA, has been implicated in persister formation in *E*. *coli* K12 [51] and may also play a role here. Ids gene regulation in general has been linked with the MrpJ transcriptional network important for *P*. *mirabilis* virulence [27]. The observation of increased MR/P fimbrial expression (Fig 1) suggests a potential link between Ids-induced changes, MrpJ, and changes in virulence.

More generally, we have presented evidence of a peer pressure system for recognition that iteratively winnows non-self cells from participating in the collective behavior of swarming—a social activity between cells that is observed among many bacterial species. Ids-mediated macroscale territorial behavior emerges from the sum of cell-cell contacts within a swarm [15, 17, 20, 52]. In this Ids model, cells do not receive any information about population composition and behavior other than that from their immediate neighbors, which is different when compared to other examples of bacterial collective behavior. For example, in bacterial quorum sensing, secretion of diffusible small molecules into the environment provides a global tracker accessible to all individuals in a group [53, 54]. Therefore, each individual cell has potentially equal access to the external signal, because the signal molecule can freely diffuse between/among cells. Each *P*. *mirabilis* cell, however, has access only to the signal of a physically adjacent cell. As such, in Ids mismatch-mediated exclusion, any information about the swarming population as a whole is decentralized and distributed among every member of the swarm. Access to that information is restricted to clusters of adjacent, neighboring cells. In these respects, Ids-mediated control represents an orthogonal model for collective behavior in bacteria that provides new opportunities to explore cell-cell communication, especially as regards to spatial coordination. Any theoretical model of Ids-mediated behavior will likely need to differ from those describing quorum sensing of diffusible molecules.

The Ids self-recognition system has distinct qualities from other contact-associated systems that have been described as bacterial communication. CDI systems [55], for example, have been described as lethal [56] or inducing permanent persister-like states [57]. However, broad spatial analysis has yet to be more generally pursued. *P*. *mirabilis* swarms could provide an excellent framework for directly analyzing individual cells before, during, and after Ids communication and for examining the global spatial consequences to these local interactions. Tracking individual cell fates through swarming and consolidation will help in this regard. Moreover, the ability of the Ids system to temporally and spatially control non-self cells by altering cell state raises the question of which other mechanisms for contact-mediated signaling in bacteria enable sophisticated interactions between individuals.

Finally, we find it unlikely that the Ids system is a specific adaptation to mitigate antibiotic pressure. This places it in contrast with Ghosh et al [57] who recently modelled CDI-mediated persistence in *E*. *coli* as a bet-hedging mechanism. We speculate instead that the antibiotic tolerance observed in this study represents an evolutionary "spandrel" of the type described by Gould and Lewontin [58, 59]. Ids-induced antibiotic tolerance in this case would be a by-product of its primary adaptive feature, regulating clonal swarm composition. Under this view, the antibiotic tolerance data shown in this paper should be regarded as a method of measuring Ids-induced phenotypic shifts in the context of territorial exclusion.

## Materials and methods

### Media

All strains used in this study are described in Table 1. *P*. *mirabilis* strains were maintained on LSW- agar [60]. CM55 blood agar base agar (Oxoid, Basingstoke UK) was used as a swarm-permissive agar. *E*. *coli* strains were maintained on Lennox lysogeny broth (LB) agar. All liquid cultures were grown in LB broth at 37°C with shaking. Swarm plates were grown either at room temperature or at 37°C. Antibiotics were added when appropriate at the following concentrations: kanamycin 35 μg ml^-1^, chloramphenicol 50 μg ml^-1^, carbenicillin 100 μg ml^-1^, ampicillin 100 μg ml^-1^, and tetracycline 15 μg ml^-1^.

**Table 1:** Strains used in this study.

Strain (All are derived from *P*. *mirabilis* strain BB2000 unless otherwise noted.)	Description	Source
wildtype	*P*. *mirabilis* BB2000	[60]
wildtype-GFP	Wildtype constitutively producing GFPmut2	[20]
wildtype-DsRed	Wildtype constitutively producing DsRed	[20]
wildtype-_HS_	Wildtype containing vector for constitutive expression of genes *flhDC*	[69]
Δ*ids*	BB2000 Δ*ids*::*Tn-Cm(R)*	[20]
Δ*ids-*GFP	Δ*ids* constitutively expressing GFPmut2	[20]
Δ*ids*-_HS_	Δ*ids* containing vector for constitutive expression of genes *flhDC*	[69]
Δ*idsE*	BB2000 with a chromosomal Δ*idsE* deletion	[21]
CCS01	Δ*ids* complemented by a vector containing the *ids* operon under its native promoter	[17, 20]
CCS02	Δ*ids* complemented by a vector containing the *ids* operon, where the variable region of *idsD* was replaced with that of the variable region of *P*. *mirabilis* strain HI4320	[16]
CCS06	Δ*ids* complemented by a vector containing genes *idsABCDF* under the native promoter	[17]
MJT01	A chromosomal Δ*idsE* deletion strain where the type VI secretion is inactivated by mutation of gene *tssB/vipA* [39]	This study
MJT02	Δ*ids* with a chromosomal Venus fluorescent protein reporter immediately downstream of gene *BB2000_0531*	This study
SM10λpir	*E*. *coli* cloning and mating strain for moving vectors into *P*. *mirabilis*	[70]
MFDpir	Mu-free *E*. *coli* cloning and mating strain for moving vectors into *P*. *mirabilis*	[62]
Δ*idsE*Δ*relA*	BB2000 background with Δ*idsE*Δ*relA* gene deletions	This study
Δ*idsE*Δ*spoT*	BB2000 background with Δ*idsE*Δ*spoT* gene deletions	This study
Δ*idsE*Δ*relA*Δ*spoT*	BB2000 background with Δ*idsE*Δ*relA*Δ*spoT* gene deletions	This study

### Strain construction

All chromosomal mutations in BB2000 and the Δ*ids* strain were made as described in [17] with the following modifications for strains constructed *de novo* in this study: the suicide vector was pRE118 [61] and the conjugative *E*. *coli* strain was strain MFDpir [62]. The *BB2000_0531* transcriptional reporter strain MJT02 includes a gene encoding the Venus fluorescent protein [44] immediately following the stop codon of gene *BB2000_0531*. All chromosomal mutations were confirmed by PCR amplification followed by Sanger sequencing of the amplified product (Genewiz, South Plainfield NJ) or by whole genome sequencing as described in [17].

### Co-swarming experiments

Strains were grown overnight at 37°C in LB broth with appropriate antibiotics. Overnight cultures were diluted in LB broth to an optical density at 600 nm (OD_600_) of 1.0, then mixed to the desired experimental ratio and inoculated with an inoculation needle onto a CM55 swarm agar plate. Plates were incubated at 37°C for 18 hours, ensuring that the swarm had covered most of the agar plate. After incubation, swarm composition was measured by using a 48-pin multi-blot replicator to sample the swarm and replica plate on non-swarming LSW- agar with relevant antibiotics as described in [15].

### RNA-Seq

Strains were grown on swarm-permissive agar plates with appropriate antibiotics at 37°C. For consolidating cell samples of wildtype, swarm colonies were left to progress overnight and confirmed to be in consolidation phase by light microscopy. Cells from the swarm edge were then harvested by scraping with a plastic loop into 1 ml of RNA Protect solution (Qiagen, Hilden, Germany). The Δ*idsE* and CCS02 samples were harvested after overnight incubation by scraping whole colonies into 1 ml RNA Protect solution. Total RNA was isolated using a RNeasy Mini kit (Qiagen, Hilden, Germany) according to the manufacturer’s instructions. RNA purity was measured using an Agilent 2200 Tapestation (Agilent, Santa Clara, CA). To enrich mRNA, rRNA was digested using terminator 5’ phosphate dependent exonuclease (Illumina, San Diego, CA) according to the manufacturer’s instructions. Enriched RNA samples were purified by phenol-chloroform extraction [63].

The cDNA libraries were prepared from mRNA-enriched RNA samples using an NEBNext Ultra RNA library prep kit (New England Biolabs, Ipswich, MA) according to the manufacturer’s instructions. Libraries were sequenced on an Illumina HiSeq 2500 instrument with 250 bp single-end reads, and base-calling was done with Illumina CASAVA 1.8 in the Harvard University Bauer Core Facility. Sequences were matched to BB2000 reference genome PMID: 24009111 (accession number CP004022) using TopHat2 using default arguments [64]. Differential expression data were generated using the Cufflinks RNA-Seq analysis suite [65] run on the Harvard Odyssey cluster. Specifically, the mRNA abundance data were generated using Cufflinks 2.1.1 with max-multiread-fraction 0.9 and -multi-read-correct. Samples were combined using cuffmerge with default arguments. Differential expression data were generated using Cuffdiff 2.1.1 with total-hits-norm. The data was analyzed using the CummeRbund package for R and Microsoft Excel. Gene functions were taken from the KEGG and COG databases [66, 67]. The data shown in this paper represent the combined analysis of two independent biological and are available at NCBI GEO accession number GSE131647.

### Fluorescent-activated cell sorting

Samples of excluded Δ*ids* cells were obtained through fluorescent-activated cell sorting (FACS). Fluorescent strains of wildtype-DsRed and Δ*ids*-GFP were grown in liquid at 37°C overnight and normalized to OD_600_ 1.0. Cultures were then mixed to the desired experimental ratio and spotted on swarm agar. After the emergence of the third swarm ring, swarm colonies were harvested into 1X phosphate-buffered saline (PBS) and sorted using a BD FACSAria cell sorter (BD Biosciences, San Jose, CA) into RNA-Protect solution. cDNA samples for RNA-Seq were prepared from sorted samples as described above.

### Microscopy

For experiments where strain ratio during swarming was examined, liquid cultures were grown overnight with shaking at 37°C. Cultures were normalized to OD_600_ 1.0, mixed to the desired experimental ratio, then used to inoculate swarm-permissive agar plates, which were incubated at room temperature overnight at room temperature to allow inoculum development. Plates were then imaged at 30-minute intervals, incubating at 37°C between measurements. For experiments with the *BB2000_0531* transcriptional reporter, 1 μl of mixed, normalized overnight culture were used to inoculate a 1-mm swarm agar pad, which was incubated at 37°C for four hours prior to imaging. Images were taken in GFP (150 ms exposure), RFP (500 ms exposure), and phase contrast channels using a Leica DM5500B microscope (Leica Microsystems, Buffalo Grove IL) and CoolSnap HQ CCD camera (Photometrics, Tucson AZ) cooled to -20°C. MetaMorph version 7.8.0.0 (Molecular Devices, Sunnyvale CA) was used for image acquisition, and FIJI [68] was used for image analysis. Raw images are available on Open Science Framework.

### Antibiotic tolerance assay

Strains were grown on swarm-permissive agar plates at 37°C until swarms reached the second round of consolidation (approximately six hours). Swarm colonies were harvested into LB broth and diluted in LB broth to OD_600_ 1.0. Prior to antibiotic exposure, a sample was taken, serially diluted in LB broth, and plated on LSW- agar to count colony-forming units (CFUs/ml) in the sample. Antibiotics were added to the normalized culture at the following concentrations: ampicillin 100 μg ml^-1^, kanamycin 60 μg ml^-1^, streptomycin 50 μg ml^-1^, and ciprofloxacin 1 μg ml^-1^. Each mixture was incubated with shaking at 37°C. At the specified time-points, samples were taken, serially diluted in LB broth and plated on LSW- agar to measure CFUs/ml. LSW- agar plates were incubated for 16 hours or until visible colonies appeared. Colonies were counted using FIJI. Experiments were performed in triplicate.

### Growth recovery assay following territorial exclusion

Co-swarms of Δ*ids-*GFP and wildtype-DsRed were inoculated onto CM55 plates and allowed to swarm at 37°C. Samples were harvested from swarm plates and sorted via FACS as described above, except cells were sorted into PBS solution. Immediately after sorting, portions of sorted cell suspension for each strain, containing equal numbers of sorted particles, were used as inoculum for overnight cultures grown at 37°C with shaking in a Tecan Infinite 200 Pro microplate reader (Tecan, Männedorf, Switzerland). OD_600_ measurements were taken hourly. Experiments were performed in triplicate. For experiments with strains CCS01 and CCS02, cell sorting was unnecessary. Instead, clonal swarms were directly harvested into PBS. The resulting suspension was diluted to OD_600_ 0.1 and used as inoculum for cultures containing relevant antibiotics.

### ppGpp quantification using high pressure liquid chromatography (HPLC)

A high-performance liquid chromatography (HPLC)-based method was used to quantify ppGpp levels, based on the work of Varik et al [42]. Swarm colonies of *P*. *mirabilis* were grown to the second swarm ring on CM55 agar. Samples for chromatography were obtained by harvesting cells in 1 ml 1M acetic acid and immediately flash-freezing in liquid nitrogen. Samples were thawed on ice for 1 hour 30 minutes with occasional vortexing, freeze-dried overnight and resuspended in 200 μl MQ-H_2_O, and then centrifuged at 4°C for 30 min to remove any insoluble fragments. Supernatants were run on a Spherisorb strong ion exchange chromatography column (80 Å, 4.6 by 150 mm, 5 μm, Waters, Milford MA). An isocratic program was used with flow rate 1.5 ml/min in running buffer consisting of 0.36 M ammonium dihydrogen phosphate, 2.5% acetonitrile (v/v), pH 3.6. Nucleotide concentrations were quantified by measuring UV absorbance at 252 nm, comparing peaks to those obtained from purified nucleotide and ppGpp samples (Trilink Biotechnologies, San Diego, CA).

## Supporting information

S1 Fig Disruption of the Type VI secretion system removes Ids-mediated antibiotic tolerance. Cells were harvested from clonal swarm plates and exposed to 100 μg ml^-1^ ampicillin. After 12 hours, viable cells were calculated from growth on fresh LSW- agar. Strains shown are the wildtype, Δ*ids*, and MJT01 strains. Strain MJT01 is an Δ*idsE*-derived strain containing a non-functional type VI secretion system [39] and so does not secrete IdsD. The deletion of *idsE* and the disruption of the T6SS-encoding gene, *tssB/vipA*, are unmarked and nonpolar on the chromosome. Each of three biological replicates is presented.
(EPS)

S2 Fig Growth in liquid disrupts Ids-mediated antibiotic tolerance. Killing curve of wildtype, Δ*ids* and Δ*idsE* strains. Cells were isolated from stationary phase cultures, exposed to 100 μg ml^-1^ ampicillin, and assessed for viability at each indicated timepoint. Viable cells were calculated after growth on fresh LSW- agar. Each of three biological replicates is presented.
(EPS)

S3 Fig Clonal Ids mismatch mutant strains do not have long-term growth defects. Cells were harvested from swarm plates and inoculated into fresh LB media with antibiotics. Growth at 37°C was assessed by optical density at 600 nm (OD_600 nm_). Strain CCS01 is a Δ*ids* strain complemented by a vector encoding the native *ids* operon and is considered a clonal self population. Strain CCS02, which is an Ids mismatch strain, expresses an IdsE protein unable to bind the incoming IdsD proteins. Strain CCS02 is the Δ*ids* background containing a plasmid expressing the *ids* operon under its native promoter; mutations to the *idsD* gene were made in the plasmid-based allele. Three biological repeats were performed. Error bars are standard deviations.
(EPS)

S4 Fig HPLC chromatograms of cell extracts illustrating detection of elevated ppGpp levels. Representative chromatograms showing UV absorbance (252 nm) over time of a SAX-HPLC column run. A) Peaks from 2 mmol nucleotide standards (ATP, CTP, GTP, UTP) and 0.5 mmol ppGpp. B) Peaks from running purified nucleotides from wildtype (cyan) and the Δ*idsE* strain (magenta) samples purified from swarm colonies. Inset shows the peak corresponding to absorbance from ppGpp alarmone molecule.
(EPS)

S5 Fig Characterization of *relA* and *spoT* deletion mutant strains in the *idsE* background. A) Mean ppGpp absorbances from wildtype, Δ*idsE*Δ*relA*, Δ*idsE*Δ*spoT* and Δ*idsE*Δ*relA*Δ*spoT* strains, calculated by integrating areas under peaks. Three biological repeats were performed. Error bar show standard deviations. B) Swarm expansion of Δ*idsE*Δ*relA*, Δ*idsE*Δ*spoT* and Δ*idsE*Δ*relA*Δ*spoT* strains carrying empty kanamycin resistance vectors when co-swarmed with wildtype, measured through the replica plating assay described in Materials and Methods. Three biological repeats were performed.
(EPS)

S6 Fig The transcriptional shift observed in cells experiencing Ids mismatch during swarm-swarm encounters occurs prior to visual boundary formation. A time-course graph showing mean swarm fluorescence intensity over time for two conditions: Δ*ids* carrying a chromosomal BB2000_0531 fluorescence reporter (strain MJT02) swarmed towards wildtype (MJT02:wildtype, cyan squares) or the Δ*ids* strain (MJT02:Δ*ids*, gray circles). Three biological repeats were performed. Fluorescence was measured over equivalent areas for each experimental condition. Error bars are standard deviations; a.u. means arbitrary units.
(EPS)

S7 Fig Representative images of consolidating and swarming *P*. *mirabilis*. Representative phase contrast (top) and GFP fluorescence (bottom) images of swarming (left) and consolidating (right) GFP-expressing *P*. *mirabilis* taken from the time-course described in S5 Fig. Cells were motile in the actively swarming colony and immobile in the consolidating colony. Note that average cell length is longer in the actively swarming colony. Scale bars = 10 μm.
(TIF)

S8 Fig Lysis does not contribute to territorial exclusion. A) A time-course graph showing percentage of the field of view occupied by consolidating Δ*ids*-GFP under two co-swarm conditions: Δ*ids*-GFP:wildtype and Δ*ids-*GFP:Δ*ids*. B) A time-course graph showing the percentage of the swarm area occupied by Δ*ids*-GFP under Δ*ids*-GFP:wildtype and Δ*ids-*GFP:Δ*ids* co-swarm conditions. Three biological repeats were performed. Error bars show standard deviation.
(EPS)

S9 Fig Hyperswarming Δ*ids* escapes Ids-mediated territorial exclusion. Co-swarms were inoculated at the indicated ratios of wildtype and the Δ*ids* strain as well as derived variants that do not consolidate (i.e., hyperswarm). Formation of hyperswarmer lineages, denoted with a subscript “HS,” was due to constitutive *in trans* expression of *flhDC* as previously described [28]. (Left) The Δ*ids* strain was co-swarmed with wildtype-_HS_. (Middle) The Δ*ids-*_HS_ strain was co-swarmed with wildtype-_HS_. (Right) The Δ*ids*-_HS_ strain was co-swarmed with wildtype-_HS_. Co-swarms were assessed for spatial distribution as detailed in the main text. Y-axis shows maximum swarm distance of the Δ*ids* strain as a ratio of the swarm distance for the wildtype (right) or wildtype-derived strains (left and middle). Each of three biological replicates is presented.
(EPS)

S1 Table Significantly differentially regulated genes between wildtype and clonal Δ*ids*. (PDF)

S2 Table Significantly differentially regulated genes between wildtype and co-swarmed Δ*ids*. (PDF)

S3 Table Significantly differentially regulated genes between CCS01 and CCS06. (PDF)

S4 Table Significantly differentially regulated genes between CCS01 and CCS02. (PDF)

S5 Table Annotated (taken from KEGG and COGG databases) functions of the 35 genes for which relative transcript abundance was significantly different in co-swarmed Δ*ids*, clonal CCS02 and clonal CCS06 swarms from a swarm of a clonal wild-type population. (PDF)
