# Supplementary figures and images for: Peer pressure from a Proteus mirabilis self-recognition system controls participation in cooperative swarm motility
Source: PLoS Pathog. 2019 Jul 19;15(7):e1007885. doi: 10.1371/journal.ppat.1007885 (PMC6682164; doi:10.1371/journal.ppat.1007885)

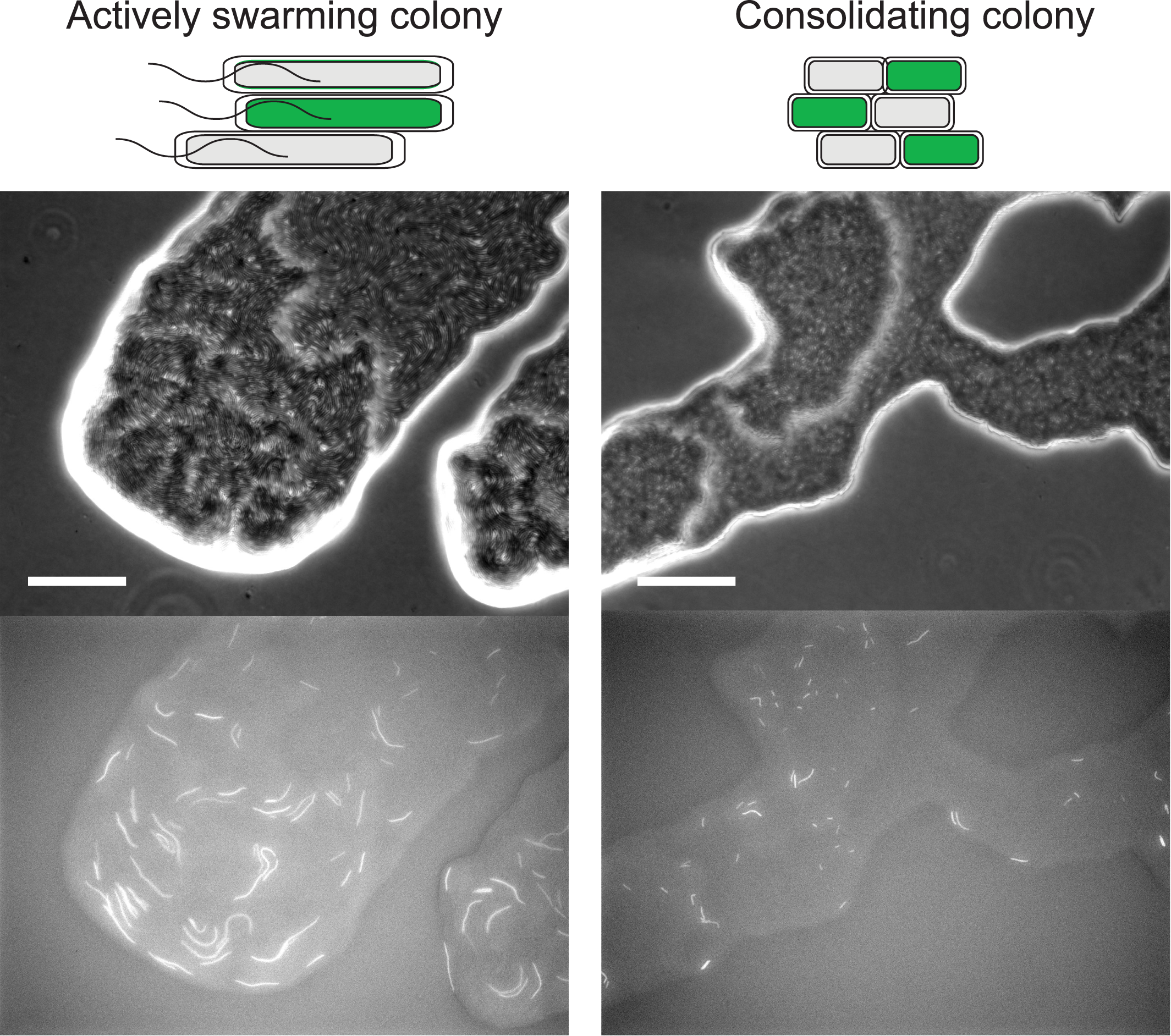

Supplement: S7 Fig — Representative phase contrast (top) and GFP fluorescence (bottom) images of swarming (left) and consolidating (right) GFP-expressing P. mirabilis taken from the time-course described in S5 Fig. Cells were motile in the actively swarming colony and immobile in the consolidating colony. Note that average cell length is longer in the actively swarming colony. Scale bars = 10 μm. (TIF) [file ppat.1007885.s007.tif]
