# Supplementary material for: Peer pressure from a Proteus mirabilis self-recognition system controls participation in cooperative swarm motility
Source: PLoS Pathog. 2019 Jul 19;15(7):e1007885. doi: 10.1371/journal.ppat.1007885 (PMC6682164; doi:10.1371/journal.ppat.1007885)
Supplement: S1 Table — (PDF) [file ppat.1007885.s010.pdf]

Supplementary Table 1. Significantly differentially regulated genes between wildtype and clonal Δ*ids*

$\log_2$ fold change	BB2000 gene name	product
2.25321	*BB2000_0110*	short hypothetical protein
8.97778	*BB2000_3003, BB2000_3004, BB2000_3005, BB2000_3006, BB2000_3007*	Ids proteins
-2.68561	*BB2000_1880*	glucose dehydrogenase
2.31615	*BB2000_2655*	short hypothetical protein
1.5191	*BB2000_2815*	hypothetical protein
2.37131	*BB2000_3145*	50S ribosomal protein
