# Supplementary material for: Peer pressure from a Proteus mirabilis self-recognition system controls participation in cooperative swarm motility
Source: PLoS Pathog. 2019 Jul 19;15(7):e1007885. doi: 10.1371/journal.ppat.1007885 (PMC6682164; doi:10.1371/journal.ppat.1007885)
Supplement: S2 Table — (PDF) [file ppat.1007885.s011.pdf]

Supplementary Table 2. Significantly differentially regulated genes between wildtype and co-swarmed Δ*ids*

log_2 fold change	BB2000 gene name	product
-7.62439	*BB2000_0139*	hypothetical protein
-7.52894	*BB2000_0110*	hypothetical protein
-7.00039	*idrA*	IdrA
-6.79654	*zapD*	type I secretion outer membrane protein
-6.79654	*zapC*	type I secretion protein
-6.79654	*zapB*	Type I secretion ATP-binding protein
-6.79654	*zapA*	metalloprotease
-6.79654	*zapE*	metalloprotease
-6.79654	*BB2000_0432*	metalloprotease
-6.79654	*BB2000_0433*	metalloprotease
-6.70601	*BB2000_1555*	hypothetical protein
-6.4165	*ndpA*	nucleoid-associated protein NdpA
-6.4165	*BB2000_0942*	hypothetical protein
-6.20813	*gshA*	glutamate--cysteine ligase
-6.14722	*nagC*	N-acetylglucosamine regulatory protein
-6.09512	*mgtC*	Mg(2+) transport ATPase protein C
-6.09512	*BB2000_1771*	hypothetical protein
-6.09512	*BB2000_1772*	hypothetical protein
-6.07724	*BB2000_0138*	tetrapyrrole methylase

-5.95337	*BB2000_1050*	hypothetical protein
-5.95337	*BB2000_1594*	hypothetical protein
-5.95337	*fliE*	flagellar hook-basal body complex protein
-5.91701	*BB2000_0943*	helicase
-5.85666	*BB2000_1130*	putative sulfate transporter YchM
-5.85666	*fumC*	fumarate hydratase
-5.81357	*BB2000_1316*	hypothetical protein
-5.78566	*holD*	DNA polymerase III subunit psi
-5.78566	*rplM*	50S ribosomal protein L13
-5.7783	*fliA*	flagellar biosynthesis sigma factor
-5.65649	*BB2000_0232*	hypothetical protein
-5.39259	*BB2000_0858*	CoA-binding protein
-5.23493	*flgN*	flagella synthesis protein
-5.16936	*BB2000_1925*	hypothetical protein
-5.14772	*gltX*	glutamyl-tRNA synthetase
-5.13845	*BB2000_1967*	alpha-2-macroglobulin-like lipoprotein (endopeptidase inhibitor)
-5.13845	*intB*	prophage integrase
-5.13845	*rpsB*	30S ribosomal protein S2
-5.13845	*BB2000_0595*	esterase
-5.12694	*adhC*	alcohol dehydrogenase (glutathione-dependent formaldehyde dehydrogenase)
-5.06481	*sucA*	2-oxoglutarate dehydrogenase E1 component

-5.06481	*sucB*	dihydrolipoamide succinyltransferase component of 2-oxoglutarate dehydrogenase complex
-5.06481	*sucC*	succinyl-CoA synthetase subunit beta
-5.06481	*sucD*	succinyl-CoA synthetase alpha chain
-5.06481	*atpI*	F0F1 ATP synthase subunit I
-5.06481	*pheS*	phenylalanyl-tRNA synthetase alpha chain
-5.06481	*ihfA*	integration host factor subunit alpha
-5.05064	*alaS*	alanyl-tRNA synthetase
-5.0021	*ogt*	methylated-DNA--protein-cysteine methyltransferase
-5.0021	*cspE*	cold shock protein CspE
-5.0021	*BB2000_0974*	hypothetical protein
-4.91336	*fabH*	3-oxoacyl-[acyl-carrier-protein] synthase III
-4.91336	*fabD*	malonyl CoA-acyl carrier protein transacylase
-4.89773	*fabG*	3-ketoacyl-(acyl-carrier-protein) reductase
-4.89773	*flhC*	transcriptional activator FlhC
-4.89773	*BB2000_0972*	lipoprotein
-4.89773	*BB2000_0973*	Maf-like protein
-4.89773	*BB2000_2655*	hypothetical protein
-4.89773	*fliZ*	flagella biosynthesis protein FliZ
-4.89383	*rpsA*	30S ribosomal protein S1
-4.85221	*prsA*	ribose-phosphate pyrophosphokinase

-4.85221	*dps*	DNA starvation/stationary phase protection protein Dps
-4.81885	*ompA*	outer membrane protein A
-4.79169	*ahpC*	alkyl hydroperoxide reductase
-4.78788	*atpB*	ATP synthase A chain
-4.78437	*lrp*	leucine-responsive transcriptional regulator
-4.78025	*rpsI*	30S ribosomal protein S9
-4.77071	*fliC1*	flagellin 1
-4.76033	*ribH*	6,7-dimethyl-8-ribityllumazine synthase
-4.69192	*BB2000_2426*	hypothetical protein
-4.65181	*znuA*	high-affinity zinc transporter periplasmic component
-4.57981	*ci*	phage repressor
-4.54869	*ptsI*	phosphoenolpyruvate-protein phosphotransferase
-4.5171	*BB2000_0948*	membrane-associated phosphatase
-4.39245	*BB2000_1470*	hypothetical protein
-4.38516	*thrS*	threonyl-tRNA synthetase
-4.38072	*tsf*	elongation factor Ts
-4.37973	*BB2000_1689*	branched chain amino acid transport protein
-4.36883	*arsR*	arsenical resistance operon repressor
-4.32908	*umoA*	upregulatior of flagellar operon (exported protein)
-4.26049	*BB2000_0690*	hypothetical protein
-4.24049	*budA*	alpha-acetolactate decarboxylase

-4.23931	*BB2000_2794*	transposase/plasmid-related protein
-4.2341	*rpoC*	DNA-directed RNA polymerase subunit beta'
-4.18377	*rplJ*	50S ribosomal protein L10
-4.17258	*rplA*	50S ribosomal protein L1
-4.1715	*rplK*	50S ribosomal protein L11
-4.15863	*secE*	preprotein translocase subunit SecE
-4.15863	*tufB*	elongation factor Tu
-4.15863	*fusA*	elongation factor G (EF-G)
-4.15863	*rpsG*	30S ribosomal protein S7
-4.15863	*BB2000_2806*	intracellular sulfur oxidation protein
-4.15863	*BB2000_2807*	intracellular sulfur oxidation protein
-4.15863	*BB2000_2808*	intracellular sulfur oxidation protein
-4.15863	*BB2000_2809*	hypothetical protein
-4.15863	*BB2000_1903*	acetyltransferase
-4.15863	*BB2000_1904*	lipoprotein
-4.15863	*BB2000_1905*	hypothetical protein
-4.15863	*BB2000_1906*	hypothetical protein
-4.15863	*BB2000_1907*	hypothetical protein
-4.15863	*aroC*	chorismate synthase
-4.15863	*BB2000_1909*	methylase
-4.15863	*ppiB*	peptidyl-prolyl cis-trans isomerase B (rotamase B)
-4.13795	*gltS*	sodium/glutamate symport carrier protein
-4.13795	*lysS*	lysyl-tRNA synthetase

-4.13795	*prfB*	peptide chain release factor 2
-4.13795	*recJ*	single-stranded-DNA-specific exonuclease
-4.13795	*dsbC*	thiol:disulfide interchange protein
-4.13795	*xerD*	tyrosine recombinase
-4.13795	*groS*	10 Kda chaperonin
-4.13728	*fxsA*	membrane protein FxsA (suppressor of F exclusion of phage T7)
-4.12557	*degS*	serine endoprotease
-4.12505	*chrR*	chromate reductase (NADPH-dependent FMN reductase)
-4.12505	*flgB*	flagellar basal body rod protein FlgB
-4.12505	*BB2000_1770*	hypothetical protein
-4.07545	*BB2000_1773*	hypothetical protein
-4.07104	*pyrC*	dihydroorotase
-4.06999	*bssS*	biofilm formation regulatory protein BssS
-4.06997	*rcsA*	colanic acid capsular biosynthesis activation protein (LuxR-family transcriptional regulator)
-4.06997	*aroQ*	3-dehydroquinate dehydratase
-4.04508	*cfa*	cyclopropane fatty acyl phospholipid synthase
-4.00702	*fldA*	flavodoxin 1
-4.00702	*flgM*	anti-sigma28 factor FlgM
-3.98086	*asnC*	asparaginyl-tRNA synthetase
-3.96389	*BB2000_0157*	putative ABC transporter ATP-binding protein

-3.94153	*BB2000_0725*	probable transporter
-3.93312	*minE*	cell division topological specificity factor MinE
-3.93312	*minD*	cell division inhibitor MinD
-3.90589	*atpH*	F0F1 ATP synthase subunit delta
-3.904	*BB2000_0710*	MFS-family transporter
-3.904	*BB2000_2819*	methyl-accepting chemotaxis protein
-3.85925	*rluB*	23S rRNA pseudouridylate synthase B
-3.85706	*nusB*	transcription antitermination protein NusB
-3.85706	*cstA*	carbon starvation protein
-3.85706	*BB2000_1097*	hypothetical protein
-3.84328	*BB2000_1100*	fimbrial chaperone
-3.8431	*BB2000_1101*	fimbrial protein
-3.8179	*BB2000_1102*	fimbrial subunit
-3.80326	*BB2000_1103*	fimbrial protein
-3.80228	*BB2000_1104*	fimbrial protein
-3.75719	*mioC*	flavodoxin
-3.75582	*ydgA*	hypothetical protein
-3.75582	*BB2000_2388*	oxidoreductase
-3.75582	*icd*	isocitrate dehydrogenase
-3.75582	*accD*	acetyl-CoA carboxylase subunit beta
-3.75582	*budB*	acetolactate synthase
-3.75582	*BB2000_2967*	iron ABC transporter, substrate-binding protein
-3.75582	*BB2000_1436*	conjugated bile acid hydrolase

-3.75582	*lpp*	major outer membrane lipoprotein (murein-lipoprotein)
-3.73617	*BB2000_1584*	transcriptional regulator
-3.72467	*BB2000_0021*	hypothetical protein
-3.72252	*adk*	adenylate kinase
-3.71905	*BB2000_1956*	lipoprotein
-3.7133	*rpmH*	50S ribosomal protein L34
-3.71034	*hpcR*	homoprotocatechuate degradative operon repressor (MarR family transcriptional regulator)
-3.70446	*BB2000_2855*	signal sensing protein
-3.70262	*glpF*	glycerol uptake facilitator protein
-3.69822	*atpA*	F0F1 ATP synthase subunit alpha
-3.69439	*BB2000_1717*	hypothetical protein
-3.69257	*BB2000_0214*	hypothetical protein
-3.67375	*gmk*	guanylate kinase
-3.67304	*cspB*	cold shock protein
-3.67304	*BB2000_1718*	hypothetical protein
-3.66895	*BB2000_2866*	lipoprotein
-3.66758	*BB2000_1466*	hypothetical protein
-3.65291	*atpG*	F0F1 ATP synthase subunit gamma
-3.58975	*apt*	adenine phosphoribosyltransferase
-3.57451	*sdaA*	L-serine deaminase 1 (L-serine deaminase 1)
-3.57285	*BB2000_1540*	aldose 1-epimerase

-3.54524	*ribA*	GTP cyclohydrolase II
-3.5395	*BB2000_0879*	hypothetical protein
-3.48619	*emrR*	transcriptional repressor MprA
-3.48454	*rimI*	ribosomal-protein-alanine N-acetyltransferase
-3.45608	*BB2000_2516*	LysR-family transcriptional regulator
-3.43835	*BB2000_1918*	fimbrial adapter
-3.42453	*BB2000_1920*	hypothetical protein
-3.4238	*phsA*	thiosulfate reductase precursor
-3.42174	*phsC*	thiosulfate reductase cytochrome b subunit
-3.3915	*accB*	biotin carboxyl carrier protein
-3.3915	*ftsB*	cell division protein FtsB
-3.36562	*sixA*	phosphohistidine phosphatase
-3.34679	*pyrF*	orotidine-5'-phosphate decarboxylase
-3.34439	*BB2000_1334*	tetratricopeptide repeat protein
-3.34439	*BB2000_1335*	hypothetical protein
-3.33523	*bcr*	bicyclomycin/multidrug efflux system
-3.33523	*BB2000_0946*	hypothetical protein
-3.33523	*gpt*	xanthine-guanine phosphoribosyltransferase
-3.33523	*gltA*	type II citrate synthase
-3.33523	*flgC*	flagellar basal-body rod protein
-3.31976	*tesB*	acyl-CoA thioesterase
-3.30618	*rnpA*	ribonuclease P
-3.28734	*frr*	ribosome recycling factor

-3.28467	*pabA*	para-aminobenzoate synthase component II
-3.27167	*BB2000_0047*	hypothetical protein
-3.27028	*cbpA*	curved DNA-binding protein CbpA
-3.27028	*aroK*	shikimate kinase I
-3.27028	*gst*	glutathionine S-transferase
-3.27028	*udk*	uridine kinase
-3.27028	*BB2000_0057*	peptidase
-3.26396	*ddg*	cold-induced palmitoleoyl transferase
-3.26396	*BB2000_1964*	hypothetical protein
-3.26393	*sfcA*	malate dehydrogenase
-3.25276	*BB2000_0775*	hypothetical protein
-3.25114	*BB2000_1029*	hypothetical protein
-3.24303	*dnaA*	chromosomal replication initiator protein
-3.23123	*BB2000_2738*	serine protease
-3.23018	*BB2000_0243*	hypothetical protein
-3.20469	*pyrH*	uridylate kinase
-3.18766	*trmD*	tRNA (guanine-N1)-methyltransferase
-3.17968	*folA*	dihydrofolate reductase
-3.16648	*BB2000_1041*	hypothetical protein
-3.1656	*uspF*	universal stress protein F
-3.12757	*fabF*	3-oxoacyl-(acyl carrier protein) synthase II
-3.12443	*BB2000_1211*	anaerobic dimethyl sulfoxide reductase chain C
-3.1229	*BB2000_0307*	hypothetical protein

-3.10776	*apaG*	ApaG
-3.0856	*fabA*	3-hydroxydecanoyl-(acyl carrier protein) dehydratase
-3.08101	*BB2000_0850*	hypothetical protein
-3.06119	*fkpA*	FKBP-type peptidyl-prolyl cis-trans isomerase
-3.05892	*cyoA*	cytochrome o ubiquinol oxidase subunit II
-3.0251	*BB2000_2076*	hypothetical protein
-3.01739	*rpsT*	30S ribosomal protein S20
-3.00841	*fliL*	flagellar basal body-associated protein FliL
-2.98301	*hemB*	delta-aminolevulinic acid dehydratase
-2.97777	*rluD*	23S rRNA pseudouridine synthase D
-2.97132	*BB2000_0534*	hypothetical protein
-2.95374	*clpB*	protein disaggregation chaperone
-2.94654	*ispD*	2-C-methyl-D-erythritol 4-phosphate cytidylyltransferase
-2.93703	*BB2000_0041*	transposase
-2.92666	*BB2000_0171*	RTX-family protein
-2.86974	*hupB*	DNA-binding protein HU-beta
-2.86974	*ppiD*	peptidyl-prolyl cis-trans isomerase (rotamase D)
-2.86974	*BB2000_0282*	competence protein
-2.86974	*BB2000_0283*	hypothetical protein
-2.86974	*BB2000_0284*	hypothetical protein
-2.86974	*rimM*	16S rRNA-processing protein RimM

-2.86974	*uspE*	universal stress protein UspE
-2.86575	*fnr*	fumarate/nitrate reduction transcriptional regulator
-2.86514	*BB2000_1698*	hypothetical protein
-2.84702	*exbD*	biopolymer transport protein
-2.84702	*exbB*	biopolymer transport protein
-2.84702	*gloA*	lactoylglutathione lyase
-2.84019	*BB2000_2194*	phosphosugar-binding regulatory protein
-2.82923	*metK*	S-adenosylmethionine synthetase
-2.81552	*BB2000_1293*	hypothetical protein
-2.8151	*lpdA*	dihydrolipoamide dehydrogenase
-2.78386	*BB2000_1204*	hypothetical protein
-2.7747	*accC*	biotin carboxylase
-2.7747	*BB2000_1016*	cold shock protein
-2.77314	*fliF*	flagellar MS-ring protein
-2.75984	*fliI*	flagellum-specific ATP synthase
-2.75713	*fliJ*	flagellar biosynthesis chaperone
-2.74589	*fliK*	flagellar hook-length control protein
-2.73892	*BB2000_1549*	lipoprotein
-2.73624	*emrE*	methyl viologen resistance protein (ethidium resistance protein)
-2.73216	*ipk*	4-diphosphocytidyl-2-C-methyl-D-erythritol kinase

-2.73216	*lolB*	outer membrane lipoprotein LolB
-2.73216	*dusB*	tRNA-dihydrouridine synthase B
-2.73216	*BB2000_2493*	hypothetical protein
-2.73216	*BB2000_0591*	putative metalloprotease
-2.73216	*uspG1*	universal stress protein G
-2.72347	*BB2000_1478*	hypothetical protein
-2.72035	*BB2000_2138*	hypothetical protein
-2.72035	*uraA*	uracil transporter
-2.72035	*upp*	uracil phosphoribosyltransferase
-2.71567	*flgG*	flagellar basal-body rod protein (distal rod protein)
-2.7133	*BB2000_0905*	hypothetical protein
-2.71226	*modB*	molybdate ABC transporter permease protein
-2.70419	*hns*	DNA-binding protein (histone-like structuring protein)
-2.70419	*BB2000_1915*	hypothetical protein
-2.68083	*BB2000_0532*	outer membrane protein assembly complex subunit YfiO
-2.68083	*rpoE*	RNA polymerase sigma factor RpoE
-2.68083	*BB2000_1537*	hypothetical protein
-2.68083	*msrB*	peptide methionine sulfoxide reductase
-2.68083	*fadL*	long-chain fatty acid outer membrane transporter
-2.68083	*thiD*	phosphomethylpyrimidine kinase

-2.66727	*BB2000_2820*	methyl-accepting chemotaxis protein
-2.66727	*BB2000_2944*	LacI-family transcriptional regulator
-2.664	*BB2000_2945*	hypothetical protein
-2.664	*BB2000_2946*	hypothetical protein
-2.65407	*BB2000_2948*	hypothetical protein
-2.65183	*BB2000_2949*	dihydrodipicolinate synthase-family protein
-2.65156	*BB2000_2950*	hypothetical protein
-2.65156	*BB2000_0792*	hypothetical protein
-2.64773	*rplS*	50S ribosomal protein L19
-2.64171	*hpr*	phosphohistidinoprotein-hexose phosphotransferase component of PTS system (Hpr)
-2.64171	*uspA*	universal stress protein A
-2.63155	*BB2000_0146*	serine/threonine transporter SstT
-2.61527	*efp*	elongation factor P
-2.61469	*lpxD*	UDP-3-O-[3-hydroxymyristoyl] glucosamine N-acyltransferase
-2.61193	*ptsN*	PTS IIA-like nitrogen-regulatory protein PtsN
-2.60876	*sthA*	soluble pyridine nucleotide transhydrogenase
-2.58066	*flgF*	flagellar basal-body rod protein
-2.5767	*BB2000_1968*	hypothetical protein
-2.56561	*BB2000_1963*	lipoprotein
-2.56536	*acrR*	DNA-binding transcriptional repressor AcrR

-2.56536	*BB2000_0297*	cytoplasmic sulphur reductase
-2.56536	*kefA*	potassium efflux protein KefA
-2.56536	*BB2000_0938*	hypothetical protein
-2.56536	*cysS*	cysteinyl-tRNA synthetase
-2.56536	*BB2000_2598*	radical SAM superfamily protein
-2.56536	*narP*	nitrate/nitrite response regulator
-2.56415	*idrB*	IdrB
-2.56245	*BB2000_0308*	putative GTP-binding protein YjiA
-2.55841	*BB2000_0873*	hypothetical protein
-2.54851	*BB2000_1383*	hypothetical protein
-2.53444	*pyrD*	dihydroorotate dehydrogenase
-2.53386	*BB2000_1449*	hypothetical protein
-2.5311	*BB2000_1679*	lipid kinase
-2.51191	*aldB*	aldehyde dehydrogenase
-2.47086	*BB2000_1792*	hypothetical protein
-2.46225	*BB2000_1793*	acetyltransferase
-2.45942	*BB2000_1794*	NADH-dependent flavin oxidoreductase
-2.45874	*BB2000_1795*	hypothetical protein
-2.45723	*BB2000_2844*	transposase
-2.45723	*BB2000_2050*	hypothetical protein
-2.45255	*tpx*	thiol peroxidase
-2.44042	*epd*	erythrose 4-phosphate dehydrogenase
-2.43555	*glpT*	sn-glycerol-3-phosphate transporter

-2.43555	*BB2000_2321*	hypothetical protein
-2.43555	*nlpB*	lipoprotein
-2.43126	*BB2000_0757*	hypothetical protein
-2.42977	*macA*	macrolide transporter subunit MacA
-2.42959	*macB*	macrolide transporter ATP-binding /permease protein
-2.42486	*clpS*	ATP-dependent Clp protease adaptor protein
-2.42304	*clpA*	ATP-dependent Clp protease ATP-binding subunit
-2.41867	*rraA*	ribonuclease activity regulator protein RraA
-2.41395	*speG*	spermidine N(1)-acetyltransferase (diamine acetyltransferase)
-2.41068	*dnaN*	DNA polymerase III subunit beta
-2.40031	*BB2000_0476*	metallo-beta-lactamase superfamily protein
-2.39614	*BB2000_1576*	glucose 1-dehydrogenase
-2.38836	*BB2000_2175*	hypothetical protein
-2.38719	*BB2000_2176*	hypothetical protein
-2.38717	*BB2000_2177*	ArsR-family transcriptional regulator
-2.37226	*spr*	putative outer membrane lipoprotein
-2.37122	*BB2000_0951*	elongation factor P-like protein
-2.3648	*BB2000_0953*	MutT/NUDIX family protein
-2.36211	*nfo*	endonuclease IV
-2.36058	*BB2000_0955*	transposase

-2.35577	*BB2000_0950*	hypothetical protein
-2.35577	*glpQ*	glycerophosphodiester phosphodiesterase
-2.34351	*cyoE*	protoheme IX farnesyltransferase
-2.34073	*BB2000_0611*	hypothetical protein
-2.33788	*tktA*	transketolase
-2.33449	*BB2000_1459*	AsnC-family transcriptional regulator
-2.33283	*BB2000_0038*	hypothetical protein
-2.32491	*pabB*	para-aminobenzoate synthase component I
-2.32048	*BB2000_1696*	hypothetical protein
-2.31604	*dgkA*	diacylglycerol kinase
-2.31399	*BB2000_2548*	pyridoxal-dependent decarboxylase
-2.29621	*BB2000_2549*	Mg(2+)/citrate complex transporter
-2.27728	*deoB*	phosphopentomutase
-2.27158	*deoA*	thymidine phosphorylase
-2.26571	*deoC*	deoxyribose-phosphate aldolase
-2.26571	*BB2000_2554*	Na+ dependent nucleoside transporter
-2.26571	*BB2000_2555*	TatD-related deoxyribonuclease
-2.24943	*BB2000_1360*	dsRNA-binding protein
-2.24943	*BB2000_1361*	hypothetical protein
-2.24943	*BB2000_1362*	hypothetical protein
-2.24943	*mipA*	MltA-interacting protein precursor
-2.24943	*hypF*	hydrogenase maturation protein
-2.24943	*dadB*	alanine racemase, catabolic

-2.24943	*dadA*	D-amino acid dehydrogenase small subunit
-2.2345	*BB2000_2885*	hypothetical protein
-2.23393	*BB2000_2886*	plasmid-related protein
-2.20559	*aroB*	3-dehydroquinate synthase
-2.20559	*ptsO*	phosphocarrier protein
-2.20458	*BB2000_0088*	hypothetical protein
-2.19252	*pykA*	pyruvate kinase
-2.18899	*hexR*	DNA-binding transcriptional regulator HexR
-2.18837	*tolB*	translocation protein TolB
-2.18676	*pal*	peptidoglycan-associated outer membrane lipoprotein
-2.17162	*BB2000_0653*	hypothetical protein
-2.17162	*rplT*	50S ribosomal protein L20
-2.17162	*flgK*	flagellar hook-associated protein 1
-2.16662	*lexA*	LexA repressor
-2.15842	*btuC*	vtamin B12-transporter permease
-2.15842	*btuD*	vitamin B12 import ATP-binding protein
-2.15842	*arnB*	UDP-4-amino-4-deoxy-L-arabinose--oxoglutarate aminotransferase
-2.15842	*arnC*	undecaprenyl phosphate 4-deoxy-4-formamido-L-arabinose transferase

-2.15842	*arnA*	bifunctional UDP-glucuronic acid decarboxylase/UDP-4-amino-4-deoxy-L-arabinose formyltransferase
-2.15842	*BB2000_1083*	polysaccharide deacetylase
-2.15842	*arnT*	4-amino-4-deoxy-L-arabinose transferase
-2.15842	*BB2000_1085*	hypothetical protein
-2.15842	*BB2000_1086*	hypothetical protein
-2.15842	*BB2000_0398*	hypothetical protein
-2.15842	*fliD*	flagellar capping protein
-2.14383	*fliS*	flagellar protein FliS
-2.14383	*fliT*	flagella protein
-2.14383	*fabZ*	(3R)-hydroxymyristoyl-ACP dehydratase
-2.14383	*glnS*	glutaminyl-tRNA synthetase
-2.14383	*mdtK*	multidrug efflux protein
-2.14022	*BB2000_2310*	oligo-nucleotide binding protein (suppressor of ushA transcription)
-2.13881	*tgt*	queuine tRNA-ribosyltransferase
-2.1244	*cheZ*	chemotaxis regulator CheZ
-2.12014	*BB2000_2623*	hypothetical protein
-2.11964	*cheY*	chemotaxis response regulator
-2.11356	*BB2000_0376*	hypothetical protein
-2.10765	*cueR*	MerR-family transcriptional regulator (copper efflux regulator)

-2.09846	*pgk*	phosphoglycerate kinase
-2.09758	*BB2000_0108*	cytochrome d ubiquinol oxidase subunit III
-2.09758	*BB2000_1027*	hypothetical protein
-2.09758	*BB2000_1460*	LysE-type transporter
-2.09003	*BB2000_3262*	hypothetical protein
-2.05317	*BB2000_0706*	acetyltransferase
-2.04992	*BB2000_2260*	hypothetical protein
-2.03484	*caiD*	carnitinyl-CoA dehydratase
-2.02839	*BB2000_0286*	AsnC-family transcriptional regulator
-2.01944	*BB2000_1524*	hypothetical protein
-2.01214	*gntX*	gluconate metabolism protein
-2.00073	*glpE*	thiosulfate sulfurtransferase (gluconate metabolism protein)
-1.99776	*glpG*	intramembrane serine protease GlpG
-1.99762	*glpR*	DNA-binding transcriptional repressor GlpR
-1.99241	*metF*	5,10-methylenetetrahydrofolate reductase
-1.99127	*corC*	magnesium and cobalt efflux protein
-1.98928	*BB2000_2854*	insulinase (Peptidase family M16)
-1.96765	*rbsC*	ribose ABC transporter permease protein
-1.96678	*rbsA*	D-ribose transporter ATP binding protein
-1.96627	*rbsD*	high affinity ribose transport protein
-1.94616	*BB2000_0747*	hypothetical protein
-1.94616	*terE*	tellurite resistance protein

-1.92467	*terB*	tellurite resistance protein
-1.91238	*terZ*	tellurite resistance protein
-1.91238	*idrD*	IdrD
-1.90014	*focA*	probable formate transporter
-1.89467	*BB2000_0779*	hypothetical protein
-1.88659	*ppx*	exopolyphosphatase
-1.8865	*accA*	acetyl-CoA carboxylase carboxyltransferase subunit alpha
-1.88624	*BB2000_2273*	hypothetical protein
-1.8819	*BB2000_1829*	phage antitermination protein
-1.87908	*glyQ*	glycyl-tRNA synthetase subunit alpha
-1.87817	*BB2000_0738*	glutathione S-transferase
-1.86296	*dcd*	deoxycytidine triphosphate deaminase
-1.85934	*cutC*	copper homeostasis protein CutC
-1.85443	*BB2000_1135*	chaperone
-1.84853	*ompF*	outer membrane porin
-1.8455	*maeB*	malic enzyme
-1.84089	*BB2000_0317*	TetR-family transcriptional regulator
-1.83993	*ssb*	single-strand binding protein
-1.83813	*kefB*	glutathione-regulated potassium-efflux system protein KefB
-1.83813	*kefG*	glutathione-regulated potassium-efflux system ancillary protein KefG

-1.83813	*recF*	recombination protein F
-1.83813	*mreB*	rod shape-determining protein MreB
-1.83813	*mreC*	rod shape-determining protein MreC
-1.83813	*mreD*	rod shape-determining protein MreD
-1.82119	*BB2000_0077*	inhibitor of septum formation
-1.81957	*cafA*	ribonuclease G
-1.81957	*BB2000_0079*	hypothetical protein
-1.81957	*BB2000_0080*	carbon-nitrogen hydrolase
-1.81957	*tldD*	protease TldD
-1.81957	*BB2000_0082*	exported ribonuclease
-1.81653	*BB2000_0083*	ribonuclease inhibitor
-1.80129	*thiB*	thiamine ABC transporter, substrate-binding protein
-1.80099	*thiP*	thiamine transporter membrane protein
-1.79931	*thiQ*	thiamine transporter ATP-binding subunit
-1.7925	*BB2000_2464*	Possible excisionase
-1.79092	*BB2000_2465*	hypothetical protein
-1.78825	*rapA*	ATP-dependent helicase HepA
-1.78621	*rpoS*	RNA polymerase sigma factor RpoS
-1.78468	*can*	carbonic anhydrase
-1.78468	*BB2000_0326*	hypothetical protein
-1.77257	*BB2000_1694*	hypothetical protein
-1.77257	*wzz*	ferric enterobactin transport protein FepE

-1.77257	*BB2000_1844*	lipoprotein
-1.77079	*BB2000_1292*	beta-eliminating lyase
-1.76717	*dcuC*	C4-dicarboxylate transporter DcuC
-1.76642	*BB2000_2098*	Z-ring-associated protein
-1.75415	*gcvR*	glycine cleavage system transcriptional repressor
-1.75003	*bcp*	thioredoxin-dependent thiol peroxidase
-1.74268	*purN*	phosphoribosylglycinamide formyltransferase (5'-phosphoribosylglycinamide transformylase)
-1.74235	*BB2000_2088*	hypothetical protein
-1.74235	*tpiA*	triosephosphate isomerase
-1.73782	*dksA*	DnaK transcriptional regulator DksA
-1.73782	*BB2000_2229*	fimbrial subunit
-1.73782	*BB2000_1892*	hypothetical protein
-1.73782	*folC*	bifunctional folylpolyglutamate synthase/ dihydrofolate synthase
-1.73141	*secF*	preprotein translocase subunit SecF
-1.72278	*BB2000_2389*	hypothetical protein
-1.72278	*pepB*	aminopeptidase B
-1.72278	*BB2000_0145*	hypothetical protein
-1.72088	*dut*	deoxyuridine 5'-triphosphate nucleotidohydrolase
-1.72003	*BB2000_0869*	LysR-family transcriptional regulator
-1.70778	*srmB*	ATP-dependent RNA helicase SrmB
-1.70778	*BB2000_0048*	hypothetical protein

-1.70458	*potD*	spermidine/putrescine ABC transporter periplasmic substrate-binding protein
-1.70232	*potC*	spermidine/putrescine ABC transporter membrane protein
-1.70116	*potB*	spermidine/putrescine ABC transporter membrane protein
-1.69845	*potA*	putrescine/spermidine ABC transporter ATPase protein
-1.69527	*dapA*	dihydrodipicolinate synthase
-1.69395	*purU*	formyltetrahydrofolate deformylase
-1.69106	*rseA*	anti-RNA polymerase sigma factor SigE
-1.68596	*BB2000_1438*	peptidoglycan-binding protein
-1.68552	*sufE*	cysteine desulfuration protein
-1.68552	*sufS*	bifunctional cysteine desulfurase/selenocysteine lyase
-1.68552	*sufD*	cysteine desulfurase activator complex subunit SufD
-1.68495	*sufC*	cysteine desulfurase ATPase component
-1.68254	*sufB*	cysteine desulfurase activator complex subunit SufB
-1.65913	*sufA*	scaffold protein for iron-sulfur cluster assembly
-1.64243	*BB2000_1446*	thioesterase
-1.63433	*pbpC*	penicillin-binding protein 1C

-1.63433	*cmk*	cytidylate kinase
-1.63347	*rnt*	ribonuclease T
1.50771	*dppF*	dipeptide transporter ATP-binding subunit
1.50771	*dppD*	dipeptide transporter ATP-binding subunit
1.50874	*BB2000_1229*	integrase/recombinase
1.51063	*BB2000_2275*	hypothetical protein
1.51063	*BB2000_2276*	hypothetical protein
1.51063	*BB2000_2277*	hypothetical protein
1.51063	*BB2000_2278*	hypothetical protein
1.51276	*BB2000_2972*	multidrug efflux protein (MFS-family transporter)
1.51999	*BB2000_2336*	isochorismatase
1.52472	*clcA*	chloride channel protein
1.52642	*BB2000_2478*	hypothetical protein
1.52819	*BB2000_1703*	universal stress protein
1.53151	*fpr*	ferredoxin-NADP reductase
1.53473	*BB2000_2216*	arylsulfatase
1.53514	*idsE3*	IdsE3
1.53514	*idsF2*	IdsF2
1.53624	*BB2000_0923*	phage protein
1.54262	*bioB*	biotin synthase
1.54262	*bioF*	8-amino-7-oxononanoate synthase
1.54262	*bioC*	biotin synthesis protein BioC
1.54262	*bioD*	dithiobiotin synthetase

1.55214	*hcr*	HCP oxidoreductase, NADH-dependent
1.55382	*BB2000_3133*	hypothetical protein
1.55731	*BB2000_2761*	hypothetical protein
1.56085	*BB2000_2169*	hypothetical protein
1.56509	*BB2000_0934*	surface polysaccharide modification acyltransferase
1.57315	*narI*	respiratory nitrate reductase 1 gamma chain
1.57581	*glnK*	nitrogen regulatory protein P-II
1.57581	*amtB*	ammonium transporter
1.58107	*BB2000_0620*	hypothetical protein
1.58107	*BB2000_0621*	hypothetical protein
1.58158	*potE*	putrescine transporter
1.58319	*BB2000_0962*	autotransporter
1.58341	*hmuR2*	hemin receptor
1.58353	*BB2000_1820*	phage protein
1.58487	*BB2000_3112*	cellulose synthase regulator protein
1.58662	*BB2000_2747*	hypothetical protein
1.58783	*mltA*	murein transglycosylase A
1.59056	*BB2000_1624*	hypothetical protein
1.59758	*BB2000_3115*	signaling protein
1.60327	*rluA*	ribosomal large subunit pseudouridine synthase
1.60394	*BB2000_2956*	ABC-transporter, permease protein
1.60726	*BB2000_2333*	hypothetical protein

1.60969	*BB2000_1592*	surface polysaccharide modification acyltransferase
1.61377	*BB2000_0624*	hypothetical protein
1.61377	*BB2000_0625*	hypothetical protein
1.61377	*BB2000_0626*	hypothetical protein
1.61392	*aslB*	Radical SAM superfamily protein (probable arylsulfatase-activating protein)
1.61605	*cysH*	phosphoadenosine phosphosulfate reductase
1.61605	*cysI*	sulfite reductase subunit beta
1.61605	*cysJ*	sulfite reductase [NADPH] flavoprotein alpha-component
1.62872	*BB2000_3100*	fimbrial chaperone
1.62925	*lysA*	diaminopimelate decarboxylase
1.63542	*BB2000_3096*	multidrug efflux protein
1.63542	*BB2000_3097*	multidrug efflux protein (MFS-family transporter)
1.6372	*BB2000_0424*	hypothetical protein
1.64605	*argG*	argininosuccinate synthase
1.64721	*BB2000_2201*	autotransporter
1.64761	*BB2000_0531*	sigma 54 modulation protein
1.65303	*BB2000_0924*	phage protein
1.6541	*BB2000_2696*	type III secretion system protein
1.65852	*fixA*	putative electron transfer flavoprotein FixA
1.65922	*BB2000_1226*	efflux protein

1.65939	*poxB*	pyruvate dehydrogenase
1.66979	*BB2000_1345*	lipoprotein
1.67495	*hisD*	histidinol dehydrogenase
1.67684	*galU*	UTP--glucose-1-phosphate uridylyltransferase
1.67764	*BB2000_2660*	hypothetical protein
1.68282	*BB2000_1037*	lipase
1.68584	*BB2000_2228*	hypothetical protein
1.68627	*fdhF*	formate dehydrogenase H
1.68753	*caiC*	putative crotonobetaine/carnitine-CoA ligase
1.69369	*BB2000_3134*	TonB-dependent receptor
1.69476	*BB2000_0319*	hypothetical protein
1.70222	*BB2000_0550*	MFS-family transporter
1.70895	*BB2000_0549*	GntR-family transcriptional regulator
1.71499	*cat*	chloramphenicol acetyltransferase
1.72376	*BB2000_0912*	hypothetical protein
1.73247	*BB2000_1344*	lipoprotein
1.74529	*atfC*	outer membrane usher protein
1.74571	*celY*	cellulase
1.74942	*agaZ*	tagatose 6-phosphate kinase
1.75094	*chbR*	DNA-binding transcriptional regulator ChbR
1.75355	*BB2000_0351*	two-component sensor kinase
1.76752	*BB2000_2242*	phage protein
1.76752	*BB2000_2243*	phage protein

1.77009	*BB2000_2258*	phage protein
1.77009	*BB2000_2259*	hypothetical protein
1.77263	*argO*	arginine exporter protein
1.77946	*BB2000_3013*	fimbrial protein
1.78956	*BB2000_0745*	transposase
1.79022	*dppB*	dipeptide transporter permease DppB
1.79054	*BB2000_3014*	hypothetical protein
1.79409	*BB2000_2339*	sodium:sulfate symporter
1.79437	*csaA*	protein secretion chaperone
1.79875	*BB2000_2913*	hypothetical protein
1.79875	*BB2000_2914*	hypothetical protein
1.79875	*BB2000_2915*	hypothetical protein
1.80386	*BB2000_2924*	haemagglutinin
1.8092	*BB2000_1091*	GntR-family transcriptional regulator
1.81084	*phoA*	alkaline phosphatase
1.81669	*dmsB*	anaerobic dimethyl sulfoxide reductase chain B
1.81771	*BB2000_1014*	hypothetical protein
1.81898	*BB2000_0477*	LysR-family transcriptional regulator
1.81995	*fhlA*	formate hydrogenlyase transcriptional activator
1.82128	*caiB*	crotonobetainyl-CoA:carnitine CoA-transferase
1.82329	*hycI*	hydrogenase 3 maturation protease
1.82329	*hyfJ*	hydrogenase-4 component J
1.82329	*hyfI*	hydrogenase-4 component I

1.82329	*hyfH*	hydrogenase 4 subunit H
1.82329	*hyfG*	hydrogenase-4 component G
1.82329	*hyfF*	hydrogenase 4 subunit F
1.82329	*hyfE*	hydrogenase 4 membrane subunit
1.82329	*hyfD*	hydrogenase 4 subunit D
1.82329	*hyfC*	hydrogenase-4 component C
1.82329	*hyfB*	hydrogenase 4 subunit B
1.82329	*hyfA*	hydrogenase-4 component A
1.82559	*BB2000_2350*	fimbrial subunit
1.82559	*BB2000_2351*	fimbrial subunit
1.83199	*BB2000_2989*	sodium:solute symporter
1.83297	*BB2000_2345*	fimbrial outer membrane usher protein
1.83318	*BB2000_1145*	ABC transporter, ATP-binding protein
1.85725	*caiT*	L-carnitine/gamma-butyrobetaine antiporter
1.8674	*BB2000_2919*	aminomethyltransferase
1.87075	*rnz*	ribonuclease Z
1.87167	*BB2000_2264*	phage protein
1.87373	*arsB*	arsenical pump membrane protein
1.87448	*BB2000_2244*	tail length tape measure protein
1.87592	*BB2000_2600*	outer membrane usher protein
1.89049	*cysG*	siroheme synthase
1.90934	*BB2000_1606*	hypothetical protein
1.91529	*BB2000_0352*	two-component response regulator

1.91746	*agaD*	N-acetylgalactosamine-specific PTS system, EIID component
1.91746	*agaW*	N-acetylgalactosamine-specific PTS system, EIIC component
1.92216	*dhaK1*	dihydroxyacetone kinase (glycerone kinase), kinase subunit
1.92216	*dhaK2*	dihydroxyacetone kinase, phosphatase subunit
1.9247	*BB2000_2694*	type III secretion system protein
1.92884	*BB2000_0916*	hypothetical protein
1.93118	*idsE2*	IdsE2
1.94082	*BB2000_2226*	fimbrial subunit
1.94351	*BB2000_0384*	TonB-dependent siderophore receptor
1.94714	*BB2000_2503*	hypothetical protein
1.95175	*BB2000_2951*	probable carbohydrate kinase
1.95512	*BB2000_1569*	hypothetical protein
1.95652	*BB2000_1825*	phage protein
1.95754	*mrpH*	fimbrial adhesin
1.96457	*BB2000_2325*	LuxR-family transcriptional regulator
1.96986	*BB2000_0389*	substrate-binding protein
1.97202	*BB2000_1495*	fimbrial adhesin
1.97556	*fixB*	electron transfer flavoprotein alpha subunit for carnitine metabolism
1.99331	*BB2000_3086*	ABC transporter, substrate-binding protein

1.99358	*BB2000_0551*	hydrolase
1.99486	*BB2000_0918*	hypothetical protein
1.99486	*BB2000_0919*	hypothetical protein
1.99486	*BB2000_0920*	hypothetical protein
1.99486	*BB2000_0921*	phage protein
1.99529	*BB2000_2960*	outer membrane protein
2.00618	*BB2000_2262*	phage lysozyme
2.00618	*BB2000_2263*	phage protein
2.01126	*BB2000_1605*	hypothetical protein
2.01222	*chbC*	N,N'-diacetylchitobiose-specific PTS system transporter subunit IIC
2.01488	*caiB*	crotonobetainyl-CoA:carnitine CoA-transferase
2.0221	*BB2000_2354*	fimbrial outer membrane usher protein
2.02402	*agaS*	tagatose-6-phosphate ketose/aldose isomerase
2.02473	*BB2000_3111*	cellulose synthase catalytic subunit [UDP-forming]
2.0306	*BB2000_0385*	decarboxylase
2.03312	*fixC*	putative oxidoreductase FixC
2.03516	*sufI*	repressor protein for FtsI
2.04216	*BB2000_0383*	siderophore biosynthesis protein
2.04267	*cueO*	multicopper oxidase
2.05196	*BB2000_1301*	hypothetical protein
2.05196	*BB2000_1302*	hypothetical protein

2.05222	*BB2000_2640*	MFS-family transporter
2.06057	*dcuB*	anaerobic C4-dicarboxylate transporter
2.07144	*copA*	copper exporting ATPase
2.07542	*narG*	respiratory nitrate reductase 1 alpha chain
2.07542	*narH*	respiratory nitrate reductase 1 beta chain
2.07542	*narJ*	respiratory nitrate reductase 1 delta chain
2.08088	*BB2000_1225*	acetyltransferase
2.08177	*BB2000_0925*	phage protein
2.08177	*BB2000_0926*	phage protein
2.08177	*BB2000_0927*	phage protein
2.08177	*BB2000_0928*	phage protein
2.08613	*fdrA*	membrane protein FdrA
2.09853	*leuC*	3-isopropylmalate dehydratase large subunit
2.1009	*BB2000_2238*	phage host specificity protein
2.11332	*BB2000_1798*	hypothetical protein
2.11332	*BB2000_1799*	branched-chain amino acid transporter
2.12178	*BB2000_3093*	amidohydrolase/metallopeptidase
2.12944	*speF*	ornithine decarboxylase
2.13091	*BB2000_2912*	transacylase
2.13422	*BB2000_0911*	hypothetical protein
2.1585	*BB2000_1227*	hypothetical protein
2.16719	*BB2000_1583*	ABC transporter, substrate-binding protein
2.1706	*BB2000_2247*	phage protein

2.17336	*BB2000_2916*	ATP-binding protein
2.17336	*BB2000_2917*	beta-ketoacyl-ACP synthase
2.17336	*BB2000_2918*	beta-ketoacyl-ACP synthase
2.18689	*mrpC*	fimbrial outer membrane usher protein
2.18839	*BB2000_1493*	aminotransferase
2.19564	*ydgI*	arginine/ornithine antiporter
2.2014	*BB2000_3114*	cellulose biosynthesis protein
2.20152	*aceB*	malate synthase A
2.20678	*BB2000_0909*	phage endopeptidase (lysis protein)
2.21343	*proW*	glycine betaine transporter membrane protein
2.21343	*proV*	glycine betaine/L-proline ABC transporter, ATP-binding protein
2.21621	*BB2000_1475*	hypothetical protein
2.21698	*speF*	ornithine decarboxylase
2.21864	*BB2000_1339*	transferase
2.22309	*BB2000_1297*	Na+/H+ antiporter
2.22437	*BB2000_2923*	holo-[acyl-carrier protein] synthase
2.2352	*BB2000_3008*	hypothetical protein
2.24032	*BB2000_2661*	LysR-family transcriptional regulator
2.24379	*BB2000_2634*	hypothetical protein
2.24379	*BB2000_2635*	hypothetical protein
2.24379	*BB2000_2636*	hypothetical protein
2.24587	*arcC*	carbamate kinase

2.24739	*leuA*	2-isopropylmalate synthase
2.2493	*BB2000_3085*	ABC transporter permease protein
2.25003	*BB2000_2428*	hypothetical protein
2.25117	*BB2000_1811*	hypothetical protein
2.25956	*BB2000_2348*	peroxidase
2.26889	*argC*	N-acetyl-gamma-glutamyl-phosphate reductase
2.30526	*mrpJ*	fimbrial operon regulator
2.30802	*BB2000_2250*	phage protein
2.30802	*BB2000_2251*	phage protein
2.30802	*BB2000_2252*	phage protein
2.30997	*BB2000_2684*	chaperone protein
2.33972	*BB2000_2693*	type III secretion system protein
2.35591	*BB2000_2683*	cell invasion protein
2.36644	*BB2000_1223*	hypothetical protein
2.36967	*BB2000_2695*	type III secretion system protein
2.38411	*BB2000_2114*	hypothetical protein
2.38411	*BB2000_2115*	toxin
2.38411	*BB2000_2116*	toxin
2.38411	*BB2000_2117*	toxin
2.39115	*BB2000_3015*	fimbrial protein
2.39577	*BB2000_0933*	phage protein
2.40318	*BB2000_2200*	hypothetical protein
2.40367	*BB2000_2576*	radical SAM superfamily protein

2.41673	*BB2000_2511*	hypothetical protein
2.43067	*BB2000_2334*	hypothetical protein
2.4338	*BB2000_2995*	PTS system, EIIBC component
2.43941	*BB2000_1239*	hypothetical protein
2.44033	*dmsA*	dimethyl sulfoxide reductase chain A
2.45218	*nirD*	nitrite reductase small subunit
2.45218	*nirB*	nitrite reductase [NAD(P)H] large subunit
2.46217	*BB2000_2697*	type III secretion system regulatory protein
2.47564	*leuO*	leucine transcriptional activator
2.47982	*ipdC*	indole-3-pyruvate decarboxylase
2.4848	*BB2000_0910*	hypothetical protein
2.52112	*BB2000_2633*	hypothetical protein
2.52749	*uca*	major fimbrial subunit
2.5278	*dmsB*	anaerobic dimethyl sulfoxide reductase chain B
2.52885	*fixB*	electron transfer flavoprotein alpha subunit for carnitine metabolism
2.52965	*BB2000_1625*	hypothetical protein
2.53102	*hisG*	ATP phosphoribosyltransferase
2.53879	*BB2000_1498*	fimbrial subunit
2.54757	*BB2000_1133*	hypothetical protein
2.55603	*BB2000_1607*	hypothetical protein
2.55603	*BB2000_1608*	hypothetical protein
2.56654	*BB2000_2921*	fatty acyl chain dehydratase

2.5684	*BB2000_2254*	head maturation protease
2.57025	*BB2000_2239*	phage tail protein
2.57025	*BB2000_2240*	phage protein
2.57958	*BB2000_0386*	pyridoxal-phosphate dependent enzyme
2.57958	*BB2000_0387*	octopine/opine/tauropine dehydrogenase
2.57982	*BB2000_0395*	hypothetical protein
2.58916	*BB2000_0913*	hypothetical protein
2.58916	*BB2000_0914*	hypothetical protein
2.58916	*BB2000_0915*	hypothetical protein
2.60024	*BB2000_0929*	phage protein
2.60024	*BB2000_0930*	phage protein
2.60024	*BB2000_0931*	phage protein
2.60024	*BB2000_0932*	phage protein
2.60317	*BB2000_2911*	hypothetical protein
2.61436	*fadD*	long-chain-fatty-acid--CoA ligase
2.62315	*BB2000_2681*	cell invasion protein
2.62367	*BB2000_2501*	demethylmenaquinone methyltransferase
2.66066	*BB2000_0388*	MFS-family transporter
2.66238	*BB2000_1256*	transport protein
2.68747	*BB2000_2255*	phage portal protein
2.68747	*BB2000_2256*	phage terminase, large subunit
2.70356	*BB2000_2269*	hypothetical protein
2.70356	*BB2000_2270*	hypothetical protein

2.71386	*fbpC*	ferric transporter ATP-binding subunit
2.72063	*BB2000_1591*	hypothetical protein
2.72724	*dmsA*	dimethyl sulfoxide reductase chain A
2.73072	*BB2000_2279*	hypothetical protein
2.73561	*BB2000_2346*	fimbrial chaperone protein
2.73766	*BB2000_2836*	hypothetical protein
2.74133	*BB2000_2353*	fimbrial chaperone protein
2.76965	*BB2000_1496*	fimbrial outer membrane usher protein
2.77722	*BB2000_3110*	hypothetical protein
2.79363	*hcp*	hydroxylamine reductase
2.80738	*BB2000_3016*	fimbrial protein
2.80824	*BB2000_0896*	hypothetical protein
2.81596	*caiA*	crotonobetainyl-CoA dehydrogenase
2.83572	*BB2000_2248*	phage protein
2.85597	*BB2000_2922*	3-oxoacyl-[acyl-carrier protein] reductase
2.88101	*fixA*	putative electron transfer flavoprotein FixA
2.88776	*BB2000_2249*	phage protein
2.96062	*cysD*	sulfate adenylyltransferase subunit 2
3.05738	*mrpG*	fimbrial subunit
3.05968	*caiA*	crotonobetainyl-CoA dehydrogenase
3.06275	*BB2000_0340*	hypothetical protein
3.11084	*BB2000_3103*	fimbrial subunit
3.12918	*BB2000_1294*	LysE-family transporter

3.13612	*BB2000_1570*	carbohydrate kinase/trancriptional regulator
3.13692	*BB2000_2343*	fimbrial subunit
3.14704	*BB2000_2355*	fimbrial subunit
3.17389	*narK*	nitrite extrusion protein (MFS-family transporter)
3.1739	*BB2000_1283*	MFS-family transporter
3.1739	*BB2000_1284*	hypothetical protein
3.19382	*pmpA*	fimbrial subunit
3.20432	*BB2000_1810*	hypothetical protein
3.22269	*BB2000_2352*	fimbrial protein
3.32801	*BB2000_0831*	hypothetical protein
3.33659	*BB2000_2253*	major capsid protein
3.33968	*BB2000_1499*	fimbrial subunit
3.44759	*BB2000_2344*	fimbrial subunit
3.49784	*BB2000_2502*	hypothetical protein
3.5195	*bioA*	adenosylmethionine-8-amino-7-oxononanoate aminotransferase
3.52405	*BB2000_2257*	phage terminase, small subunit
3.62037	*BB2000_0830*	hypothetical protein
3.64197	*BB2000_0922*	phage protein
3.64613	*BB2000_1497*	fimbrial chaperone protein
3.99141	*BB2000_1552*	hypothetical protein
4.14668	*BB2000_3158*	hypothetical protein
4.14668	*BB2000_3159*	hypothetical protein

4.82774	*BB2000_3198*	serine acetyltransferase
6.01507	*BB2000_0381*	ABC transporter, ATP-binding subunit
