# Supplementary material for: Peer pressure from a Proteus mirabilis self-recognition system controls participation in cooperative swarm motility
Source: PLoS Pathog. 2019 Jul 19;15(7):e1007885. doi: 10.1371/journal.ppat.1007885 (PMC6682164; doi:10.1371/journal.ppat.1007885)
Supplement: S3 Table — (PDF) [file ppat.1007885.s012.pdf]

Supplementary Table 3. Significantly differentially regulated genes between CCS01 and CCS06

log_2 fold change	BB2000 gene name	product
-4.5289	*BB2000_0427, BB2000_0428, BB2000_0429, BB2000_0430*	zapABCD
-4.04955	*BB2000_1015*	lipase
-3.7314	*BB2000_1500*	fimbrial operon regulator
-3.6785	*BB2000_1717*	hypothetical protein
-3.5754	*BB2000_1016*	cold shock protein
-3.49282	*flgC*	flagellar basal-body rod protein
-3.48269	*BB2000_1826*	phage protein (endopeptidase/lysis protein)
-3.47585	*ddg*	cold-induced palmitoleoyl transferase
-3.41201	*rnk*	nucleoside diphosphate kinase regulator
-3.2631	*fliZ, fliA, BB2000_1711, fliC2*	flagella biosynthesis protein FliZ, flagellar biosynthesis sigma factor, hypothetical protein, hypothetical protein
-3.17636	*BB2000_2819*	methyl-accepting chemotaxis protein
-3.09508	*fliJ*	flagellar biosynthesis chaperone
-3.07686	*bfd*	bacterioferritin-associated ferredoxin

-3.06231	*cspA*	cold shock protein
-3.05536	*cspB*	cold shock protein
-3.05119	*BB2000_2949*	dihydrodipicolinate synthase-family protein
-3.01576	*flgA*	flagella basal body P-ring formation protein
-2.9013	*BB2000_2950*	hypothetical protein
-2.88481	*BB2000_0879*	hypothetical protein
-2.87996	*fliF, fliG, fliH, fliI*	flagellar MS-ring protein, flagellar motor switch protein G, flagellar assembly protein H, flagellum-specific ATP synthase
-2.8306	*BB2000_1294*	LysE-family transporter
-2.75331	*BB2000_1827*	phage protein
-2.73139	*BB2000_1381*	outer membrane protein (attachment invasion locus protein)
-2.71296	*BB2000_2565*	hypothetical protein
-2.66101	*BB2000_3198*	serine acetyltransferase
-2.57831	*BB2000_1824*	phage protein
-2.57672	*BB2000_1033, map1*	hypothetical protein, methionine aminopeptidase
-2.5747	*BB2000_2344*	fimbrial subunit
-2.5683	*BB2000_1215, BB2000_1216*	PadR-family transcriptional regulator, hypothetical protein
-2.56305	*BB2000_0924*	phage protein
-2.52905	*flgD*	basal-body rod modification protein

-2.52684	*BB2000_1097*	hypothetical protein
-2.492	*fumC, BB2000_1316*	fumarate hydratase, hypothetical protein
-2.42215	*BB2000_0832*	Rhs-family protein
-2.40861	*BB2000_1586*	hypothetical protein
-2.36323	*BB2000_1818, BB2000_1819*	phage protein, phage protein
-2.3213	*BB2000_1470*	hypothetical protein
-2.31577	*BB2000_0342*	transcriptional regulator
-2.2976	*flhC*	transcriptional activator FlhC
-2.26955	*BB2000_2352*	fimbrial protein
-2.25484	*BB2000_1828*	phage holin (lysis protein)
-2.23795	*pbpC*	penicillin-binding protein 1C
-2.22757	*ribB*	3, 4-dihydroxy-2-butanone 4-phosphate synthase
-2.20865	*BB2000_2855*	signal sensing protein
-2.20668	*BB2000_0704*	threonine and homoserine efflux system
-2.20651	*BB2000_1829*	phage antitermination protein
-2.19849	*pagP, pagQ*	palmitoyl transferase
-2.19146	*BB2000_1815, BB2000_1816*	phage protein, phage protein
-2.19111		
-2.18995	*BB2000_1825*	phage protein
-2.17953	*BB2000_1809*	hypothetical protein

-2.17555	*sthA*	soluble pyridine nucleotide transhydrogenase
-2.16621	*hpmB*	hemolysin activator protein (two-partner secretion system accessory protein)
-2.16269	*BB2000_1584*	transcriptional regulator
-2.1596	*BB2000_2750*	hypothetical protein
-2.15855	*BB2000_0744*	hypothetical protein
-2.13634	*terA*	tellurite resistance protein
-2.11023	*BB2000_1820*	phage protein
-2.10422	*flgH*	flagellar basal body L-ring protein
-2.08224	*fliK*	flagellar hook-length control protein
-2.05511	*rplV*	50S ribosomal protein L22
-2.05341	*BB2000_3499*	lipoprotein
-2.04662	*BB2000_2477*	transposase
-2.04396	*flgB*	flagellar basal body rod protein FlgB
-2.03386	*BB2000_2249*	phage protein
-2.02962	*fliE*	flagellar hook-basal body complex protein
-2.02236	*BB2000_2246*	phage protein
-2.01885	*BB2000_3459*	hypothetical protein
-2.01871	*flgG*	flagellar basal-body rod protein (distal rod protein)
-2.01609	*BB2000_2250, BB2000_2251, BB2000_2252*	phage protein, phage protein, phage protein

-2.01388	*BB2000_1466*	hypothetical protein
-1.95844	*csaA*	protein secretion chaperone
-1.92888	*BB2000_2664, BB2000_2665*	hypothetical protein, hypothetical protein
-1.91794	*acs*	acetyl-CoA synthetase
-1.89363	*bioA*	adenosylmethionine-8-amino-7-oxononanoate aminotransferase
-1.89049	*BB2000_2274*	hypothetical protein
-1.87246	*BB2000_2310*	oligo-nucleotide binding protein (suppressor of *ushA* transcription)
-1.84937	*BB2000_2321*	hypothetical protein
-1.84847	*BB2000_0828*	Rhs-family protein
-1.84034	*BB2000_2951*	probable carbohydrate kinase
-1.82824	*BB2000_0710*	MFS transporter
-1.82255	*purE*	phosphoribosylaminoimidazole carboxylase catalytic subunit
-1.81668	*umoA*	upregulator of flagellar operon (exported protein)
-1.81097	*rplC*	50S ribosomal protein L3
-1.79966	*BB2000_2948*	hypothetical protein
-1.79606	*BB2000_2388*	oxidoreductase
-1.76481	*hpaC*	4-hydroxyphenylacetate 3-monooxygenase, reductase component

-1.75641	*BB2000_0157*	putative ABC transporter ATP-binding protein
-1.71853	*rplB*	50S ribosomal protein L2
-1.71844	*BB2000_3500*	acetyltransferase
-1.70908	*BB2000_1292*	beta-eliminating lyase
-1.68397	*ccm*	membrane protein (Ccm1 protein)
-1.67602	*rpsJ*	30S ribosomal protein S10
-1.65258	*BB2000_0156*	TonB-like protein
-1.64118	*rplE*	50S ribosomal protein L5
-1.62894	*BB2000_1465*	iron utilization protein
-1.61621	*caiE*	carnitine operon protein CaiE
-1.60387	*rplX*	50S ribosomal protein L24
-1.59794	*emrR*	transcriptional repressor MprA
-1.57655	*BB2000_1277*	ABC transporter ATP-binding protein
-1.57487	*metF*	5, 10-methylenetetrahydrofolate reductase
-1.53226	*BB2000_2947*	hypothetical protein
-1.52043	*rplN*	50S ribosomal protein L14
1.54585	*BB2000_3066*	hypothetical protein
1.55085	*BB2000_0885*	phage replication protein
1.55646	*BB2000_0619*	hypothetical protein
1.57944	*glpQ*	glycerophosphodiester phosphodiesterase
1.60273	*BB2000_1494*	fimbrial subunit
1.6642	*umoC*	upregulator of flagellar master operon

1.67492	*relE, BB2000_2873*	hypothetical protein
1.75798	*BB2000_1915*	hypothetical protein
1.76336	*bioD*	dethiobiotin synthetase
1.78261	*BB2000_1256*	transport protein
1.85284	*BB2000_0966*	hypothetical protein
1.87002	*cpxP*	periplasmic protein
1.90918	*BB2000_1900*	hypothetical protein
1.92804	*BB2000_3158*	hypothetical protein
1.92804	*BB2000_3159*	hypothetical protein
1.93113	*hupA*	DNA-binding protein HU-alpha (HU-2)
1.95145	*BB2000_1910*	ferritin-like protein
1.95615	*focA*	probable formate transporter
1.96074	*oppA*	oligopeptide ABC transporter, oligopeptide-binding protein
1.98106	*BB2000_1347*	lipoprotein
1.98458	*BB2000_3206*	hypothetical protein
2.02246		
2.0472	*hyb0*	hydrogenase 2 small subunit
2.04745	*mscL*	large-conductance mechanosensitive channel
2.05419	*BB2000_1225*	acetyltransferase
2.05792	*BB2000_3202*	hypothetical protein
2.06765	*BB2000_1476*	hypothetical protein

2.07174	*BB2000_0283*	hypothetical protein
2.07307	*BB2000_3487*	hypothetical protein
2.11788	*BB2000_3399*	hypothetical protein
2.12087	*pspC*	DNA-binding transcriptional activator PspC
2.12899	*uca*	major fimbrial subunit
2.12959	*BB2000_1346*	lipoprotein
2.13238	*BB2000_1222*	hypothetical protein
2.14666	*BB2000_3230*	hypothetical protein
2.14776	*ftnA*	ferritin
2.16803	*BB2000_0223*	hypothetical protein
2.17051	*BB2000_2079*	hypothetical protein
2.17953	*BB2000_1572*	hypothetical protein
2.18264	*holE2*	DNA polymerase III, theta subunit
2.1916	*BB2000_0090*	probable sigma(54) modulation protein
2.21484	*BB2000_2113*	hypothetical protein
2.22212	*BB2000_0850*	hypothetical protein
2.22446	*rob*	right origin-binding protein
2.23097	*BB2000_0492*	CsbD family general stress response protein
2.2349	*BB2000_0620*	plasmid stabilization proteins ParE and antitoxin CC2985

2.24108	*BB2000_2067, BB2000_2068, BB2000_2070*	hypothetical protein, hypothetical protein, hypothetical protein
2.37747	*BB2000_3243*	hypothetical protein
2.39124	*aphA*	acid phosphatase/phosphotransferase
2.39207	*tolC*	outer membrane channel protein
2.3984	*BB2000_3244*	hypothetical protein
2.42723	*grpE*	heat shock protein
2.4299	*BB2000_1552*	hypothetical protein
2.4513	*BB2000_2355*	fimbrial subunit
2.45713	*BB2000_1884*	PTS system EIIA component
2.45877	*BB2000_1140*	lipoprotein
2.47441	*BB2000_2182*	lipoprotein
2.49323	*fdhF*	formate dehydrogenase-H, selenopolypeptide subunit
2.52301	*BB2000_1553*	hypothetical protein
2.52643	*pspA*	phage shock protein PspA
2.52742	*BB2000_0891*	hypothetical protein
2.54178	*intB*	prophage integrase
2.58607	*pstS*	phosphate ABC transporter periplasmic substrate-binding protein PstS
2.58749	*mrpJ*	fimbrial operon regulator
2.60486	*asnA*	asparagine synthetase AsnA

2.6327	*BB2000_1164*	hypothetical protein
2.65299	*frdA*	fumarate reductase flavoprotein subunit
2.65335	*BB2000_0395*	hypothetical protein
2.685	*BB2000_2846*	hypothetical protein
2.74456	*pmfA*	major fimbrial subunit
2.77355	*BB2000_0873*	hypothetical protein
2.82506	*BB2000_0330*	hypothetical protein
2.87824	*BB2000_2098*	Z-ring-associated protein
2.88548	*ompW*	outer membrane protein W
2.93693	*BB2000_0670*	hypothetical protein
2.95495	*BB2000_1178*	lipoprotein
3.01194	*mrpA*	major mannose-resistant/Proteus-like fimbrial protein
3.01521	*BB2000_1970*	hypothetical protein
3.05046	*BB2000_1383, BB2000_1384*	hypothetical protein, hypothetical protein
3.07846	*BB2000_1467*	hypothetical protein
3.18681	*BB2000_3107*	hypothetical protein
3.27368	*tesB*	acyl-CoA thioesterase
3.3198	*BB2000_1473*	hypothetical protein
3.35001	*nhaA*	Na+/H+ antiporter
3.38338	*BB2000_0444*	hypothetical protein

3.40412	*BB2000_0426*	hypothetical protein
3.49895	*pmpA*	fimbrial subunit
3.52689	*grcA*	autonomous glycyl radical cofactor GrcA
3.58086	*BB2000_1718*	hypothetical protein
3.6441	*BB2000_2054*	hypothetical protein
3.66735	*BB2000_2043*	hypothetical protein
3.68306	*BB2000_3309*	hypothetical protein
3.76035	*BB2000_0622*	hypothetical protein
3.85907	*ttcA*	tRNA 2-thiocytidine biosynthesis protein
4.21471	*BB2000_1017*	heat shock protein
4.22406	*BB2000_2810*	hypothetical protein
4.60548	*BB2000_3433*	hypothetical protein
4.6334	*BB2000_2357*	fimbrial operon regulator
4.82102	*BB2000_1620*	hypothetical protein
5.30436	*BB2000_0531*	sigma 54 modulation protein
5.41104	*pheA*	bifunctional chorismate mutase/prephenate dehydratase
5.60442	*BB2000_0664*	hypothetical protein
6.55524	*BB2000_1414*	hypothetical protein
