# Supplementary material for: Peer pressure from a Proteus mirabilis self-recognition system controls participation in cooperative swarm motility
Source: PLoS Pathog. 2019 Jul 19;15(7):e1007885. doi: 10.1371/journal.ppat.1007885 (PMC6682164; doi:10.1371/journal.ppat.1007885)
Supplement: S4 Table — (PDF) [file ppat.1007885.s013.pdf]

Supplementary Table 4. Significantly differentially regulated genes between CCS01 and CCS02

**log2(fold_change)**	**BB2000 gene name**	**product**
-2.87845	*BB2000_0531*	sigma 54 modulation protein
-2.64616	*BB2000_0669*	hypothetical protein
-2.63777	*BB2000_0668*	hypothetical protein
-2.52299	*BB2000_3110*	hypothetical protein
-2.43888	*BB2000_0830*	hypothetical protein
-2.34279	*BB2000_1017*	heat shock protein
-2.22256	*BB2000_0493*	phosphodiesterase
-2.14	*xaxB*	toxin
-2.09976	*BB2000_2684*	chaperone protein
-2.05572	*BB2000_2913, BB2000_2914, BB2000_2915*	hypothetical protein, hypothetical protein, hypothetical protein
-2.03071	*BB2000_0667*	hypothetical protein
-2.02364	*BB2000_2761*	hypothetical protein
-2.01922	*BB2000_3468, BB2000_3469*	hypothetical protein
-1.9769	*BB2000_2601*	fimbrial chaperone
-1.87733	*BB2000_2343*	fimbrial subunit
-1.8413	*BB2000_0545*	hypothetical protein

-1.81923	*BB2000_2911*	hypothetical protein
-1.81253	*BB2000_1755*	hypothetical protein
-1.80435	*BB2000_1552*	hypothetical protein
-1.804	*BB2000_2920*	acyl carrier protein
-1.79517	*BB2000_2695*	type III secretion system protein
-1.79217	*BB2000_1604*	hypothetical protein
-1.78793	*BB2000_1182*	putative ABC transporter ATP-binding protein YbbL
-1.77049	*BB2000_2923*	holo-[acyl-carrier protein] synthase
-1.76485	*BB2000_2689*	type III secretion system protein
-1.76485	*BB2000_2690*	type III secretion system protein
-1.7577	*BB2000_2722*	microcompartments protein
-1.75531	*BB2000_3103*	fimbrial subunit
-1.73194	*rob*	right origin-binding protein
-1.67299	*BB2000_1199*	fimbrial subunit
-1.66935	*BB2000_0831*	hypothetical protein
-1.63938	*BB2000_2697*	type III secretion system regulatory protein
-1.63707	*rcsA*	colanic acid capsular biosynthesis activation protein (LuxR-family transcriptional regulator)
-1.63458	*BB2000_0445*	hypothetical protein
-1.6334	*BB2000_1617*	hypothetical protein
-1.63318	*BB2000_2355*	fimbrial subunit

-1.62633	*BB2000_2710*	QacE family quaternary ammonium compound efflux SMR transporter
-1.61553	*BB2000_2720*	microcompartments protein
-1.60813	*BB2000_2921*	hydroxymyristoyl-ACP dehydratase
-1.60662	*BB2000_2074*	hypothetical protein
-1.59488	*BB2000_0257*	hypothetical protein
-1.57819	*BB2000_2693*	type III secretion system protein
-1.55826	*BB2000_3389*	LysR-family transcriptional regulator
-1.55388	*BB2000_1393*	hypothetical protein
-1.55188	*BB2000_0351*	two-component sensor kinase
-1.54218	*BB2000_2709*	protein-tyrosine phosphatase
-1.54157	*terW*	tellurium resistance protein
-1.51772	*BB2000_0408*	minor fimbrial subunit
-1.51663	*BB2000_0319*	hypothetical protein
-1.51641	*BB2000_0666*	hypothetical protein
-1.51027	*BB2000_2718*	hypothetical protein
-1.51016	*BB2000_2352*	fimbrial protein
1.51177	*BB2000_1438*	peptidoglycan-binding protein
1.51282	*sufE*	cysteine desulfuration protein
1.52276	*sufS*	bifunctional cysteine desulfurase/selenocysteine lyase
1.52863	*sufD*	cysteine desulfurase activator complex subunit SufD

1.53109	*sufC*	cysteine desulfurase ATPase component
1.53146	*sufB*	cysteine desulfurase activator complex subunit SufB
1.53401	*BB2000_1822*	phage protein
1.53586	*mdh*	malate dehydrogenase
1.53703	*hupA*	DNA-binding protein HU-alpha (HU-2)
1.54541	*pykA*	pyruvate kinase
1.5471	*mutT*	nucleoside triphosphate pyrophosphohydrolase
1.55277	*modB*	molybdate ABC transporter permease protein
1.5577	*cydX*	cytochrome bd-I oxidase subunit CydX
1.55954	*BB2000_0643*	hypothetical protein
1.55955	*udk*	uridine kinase
1.56651	*sodB*	superoxide dismutase [Fe]
1.57226	*hupB*	DNA-binding protein HU-beta
1.57255	*gntY*	putative DNA uptake protein
1.57307	*BB2000_1358*	lipoprotein
1.57311	*BB2000_0955*	transposase
1.57589	*BB2000_3194*	rhodanese-like protein
1.57646	*BB2000_0297*	cytoplasmic sulphur reductase
1.58189	*gcp*	putative DNA-binding/iron metalloprotein/AP endonuclease

1.58345	*ispD*	2-C-methyl-D-erythritol 4-phosphate cytidylyltransferase
1.58716	*fabI*	enoyl-(acyl carrier protein) reductase
1.59526	*BB2000_0840*	hypothetical protein
1.5999	*cydB*	cytochrome D ubiquinol oxidase subunit II
1.60545	*BB2000_0949*	hypothetical protein
1.61565	*zapC*	type I secretion protein
1.63266	*tatB*	Sec-independent protein translocase
1.63961	*BB2000_3319*	hypothetical protein
1.64023	*pth*	peptidyl-tRNA hydrolase
1.65517	*tktA*	transketolase
1.66505	*aceF*	dihydrolipoamide acetyltransferase
1.66658	*aceE, pdhR*	pyruvate dehydrogenase subunit E1, transcriptional regulator PdhR
1.66658	*pdhR*	transcriptional regulator PdhR
1.66885	*secB*	preprotein translocase subunit SecB
1.6711	*metK*	S-adenosylmethionine synthetase
1.6776	*BB2000_0803*	hypothetical protein
1.67923	*hfq*	protein Hfq (host factor-I protein)
1.68098	*hflX*	putative GTPase HflX
1.68212	*znuA*	high-affinity zinc transporter periplasmic component
1.68503	*mutL*	DNA mismatch repair protein

1.68523	*miaA*	tRNA delta(2)-isopentenylpyrophosphate transferase
1.68578	*BB2000_2664*	hypothetical protein
1.69196	*BB2000_2665*	hypothetical protein
1.69318	*BB2000_2194*	phosphosugar-binding regulatory protein
1.69826	*dksA*	DnaK transcriptional regulator DksA
1.69955	*pgk*	phosphoglycerate kinase
1.70039	*BB2000_1964*	hypothetical protein
1.70375	*pabA*	para-aminobenzoate synthase component II
1.70508	*BB2000_0698*	transglycosylase associated protein
1.7094	*deaD*	ATP-dependent RNA helicase DeaD
1.7094	*BB2000_1261*	ABC transporter ATP-binding protein
1.71077	*BB2000_1262*	hypothetical protein
1.71077	*cspD*	cold shock-like protein
1.71188	*rnk*	nucleoside diphosphate kinase regulator
1.7122	*ftsB*	cell division protein FtsB
1.717	*BB2000_1694*	hypothetical protein
1.71725	*BB2000_1482*	hypothetical protein
1.71927	*BB2000_2053*	MFS-family transporter
1.72182	*BB2000_1029*	hypothetical protein
1.7224	*tpiA*	triosephosphate isomerase
1.72559	*hpmA*	hemolysin

1.72932	*ipk, lolB*	4-diphosphocytidyl-2-C-methyl-D-erythritol kinase, outer membrane lipoprotein LolB
1.73683	*speG*	spermidine N(1)-acetyltransferase (diamine acetyltransferase)
1.74322	*BB2000_1332*	translation initiation factor Sui1
1.75147	*cvpA*	colicin V production protein
1.75794	*atpD*	F0F1 ATP synthase subunit beta
1.7584	*priC*	primosomal replication protein N
1.75914	*BB2000_0301*	hypothetical protein
1.76165	*BB2000_0592*	PhoH-like ATP-binding protein
1.77641	*BB2000_1418*	transposase
1.77938	*exbB*	biopolymer transport protein
1.78016	*exbD*	biopolymer transport protein
1.79239	*holE2*	DNA polymerase III, theta subunit
1.79987	*rpoZ*	DNA-directed RNA polymerase omega chain
1.80206	*rseA*	anti-RNA polymerase sigma factor SigE
1.80233	*xseB*	exodeoxyribonuclease VII small subunit
1.81123	*BB2000_2564*	hypothetical protein
1.81197	*uspA*	universal stress protein A
1.81253	*BB2000_1761*	methyl-accepting chemotaxis protein
1.81253	*BB2000_0145*	hypothetical protein
1.81257	*atpC*	ATP synthase epsilon chain
1.81817	*cheZ*	chemotaxis regulator CheZ

1.82193	*slyD*	FKBP-type peptidyl-prolyl cis-trans isomerase
1.82624	*apaG*	ApaG
1.8266	*pyrG*	CTP synthetase
1.82696	*glyA*	serine hydroxymethyltransferase
1.8298	*bfr*	bacterioferritin
1.83272	*BB2000_1762*	methyl-accepting chemotaxis protein
1.83278	*infB*	translation initiation factor IF-2
1.83343	*cstA*	carbon starvation protein
1.83634	*motA*	flagellar motor protein MotA
1.83714	*deoC*	deoxyribose-phosphate aldolase
1.83996	*BB2000_1050*	hypothetical protein
1.84394	*ydfG*	NADP-dependent L-serine/L-allo-threonine dehydrogenase
1.84995	*BB2000_1308*	hypothetical protein
1.85102	*BB2000_1309*	hypothetical protein
1.85186	*rplQ*	50S ribosomal protein L17
1.85897	*oxaA*	putative inner membrane protein translocase component YidC
1.86347	*trpE*	anthranilate synthase component I
1.86582	*trpD*	anthranilate synthase component (glutamine amidotransferase)
1.8738	*trpD*	anthranilate phosphoribosyltransferase

1.87739	*trpC*	bifunctional indole-3-glycerol phosphate synthase/phosphoribosylanthranilate isomerase
1.87966	*trpB*	tryptophan synthase subunit beta
1.89228	*potC*	spermidine/putrescine ABC transporter membrane protein
1.90433	*potB*	spermidine/putrescine ABC transporter membrane protein
1.91043	*arnT*	4-amino-4-deoxy-L-arabinose transferase
1.91522	*BB2000_0591*	putative metalloprotease
1.91697	*cyoA*	cytochrome o ubiquinol oxidase subunit II
1.91926	*BB2000_1537*	hypothetical protein
1.91926	*motB*	chemotaxis protein (motility protein B)
1.91926	*BB2000_2177*	ArsR-family transcriptional regulator
1.92053	*BB2000_1846*	hypothetical protein
1.93026	*rssB*	swarming motility regulation two-component system, response regulator
1.93558	*accD*	acetyl-CoA carboxylase subunit beta
1.9375	*rplU*	50S ribosomal protein L21
1.95081	*BB2000_1827*	phage protein
1.95226	*aspC*	aromatic amino acid aminotransferase
1.9536	*BB2000_0281*	peptidylprolyl isomerase

1.95421	*chrR*	chromate reductase (NADPH-dependent FMN reductase)
1.95524	*lpp*	major outer membrane lipoprotein (murein-lipoprotein)
1.95785	*fliM*	flagellar motor switch protein FliM
1.98268	*fliN*	flagellar motor switch protein FliN
1.9859	*cfa*	cyclopropane fatty acyl phospholipid synthase
1.9866	*lexA*	LexA repressor
1.99436	*dgkA*	diacylglycerol kinase
1.99493	*accB*	biotin carboxyl carrier protein
2.00281	*BB2000_1478*	hypothetical protein
2.0063	*nlpI*	lipoprotein NlpI
2.00797	*rpsM*	30S ribosomal protein S13
2.01102	*purA*	adenylosuccinate synthetase
2.01107	*mdeA*	methionine gamma-lyase
2.01592	*rpsU, dnaG*	30S ribosomal protein S21, DNA primase
2.02025	*rpoD*	RNA polymerase sigma factor RpoD
2.04092	*BB2000_2511*	hypothetical protein
2.04263	*eno*	phosphopyruvate hydratase
2.04556	*folE*	GTP cyclohydrolase I
2.04819	*rcsB*	transcriptional regulator RcsB
2.05431	*fliT*	flagella protein
2.05532	*BB2000_3128*	glycosyltransferase

2.06105	*BB2000_0108*	cytochrome d ubiquinol oxidase subunit III
2.0649	*rplM*	50S ribosomal protein L13
2.06623	*gmk*	guanylate kinase
2.07122	*mioC*	flavodoxin
2.07218	*BB2000_2866*	lipoprotein
2.07572	*BB2000_3522*	hypothetical protein
2.08511	*BB2000_0937*	sulphatase
2.09077	*BB2000_0938*	hypothetical protein
2.10985	*rrmJ*	23S rRNA methyltransferase J
2.11269	*BB2000_3427*	hypothetical protein
2.11325	*rpsI*	30S ribosomal protein S9
2.11884	*BB2000_3455*	hypothetical protein
2.1256	*mreB*	rod shape-determining protein MreB
2.1256	*BB2000_2537*	phage lysis protein (holin)
2.1256	*bssS*	biofilm formation regulatory protein BssS
2.14183	*atpF*	F0F1 ATP synthase subunit B
2.14525	*BB2000_3426*	hypothetical protein
2.14753	*rplI*	50S ribosomal protein L9
2.14949	*BB2000_1470*	hypothetical protein
2.15527	*aroK*	shikimate kinase I
2.16602	*groL*	60 Kda chaperonin
2.17796	*fxsA*	membrane protein FxsA (suppressor of F exclusion of phage T7)

2.18807	*ccm*	membrane protein (Ccm1 protein)
2.19463	*BB2000_3025*	inorganic phosphate transporter
2.19755	*sthA*	soluble pyridine nucleotide transhydrogenase
2.19994	*grxA*	glutaredoxin 1
2.20278	*tig*	trigger factor
2.20651	*rffH*	glucose-1-phosphate thymidylyltransferase
2.22152	*terZ*	tellurite resistance protein
2.22328	*rplO*	50S ribosomal protein L15
2.22447	*BB2000_1578*	lipoprotein
2.22506	*flgL*	flagellar hook-associated protein 3 (hook-filament junction protein)
2.22624	*accA*	acetyl-coenzyme A carboxylase carboxyl transferase subunit alpha
2.22624	*thiD*	phosphomethylpyrimidine kinase
2.22624	*ribA*	GTP cyclohydrolase II
2.22686	*ompF*	outer membrane porin
2.22717	*gst*	glutathionine S-transferase
2.22872	*BB2000_1215*	PadR-family transcriptional regulator
2.23943	*BB2000_1216*	hypothetical protein
2.24308	*BB2000_0038*	hypothetical protein
2.24596	*BB2000_0879*	hypothetical protein
2.24596	*BB2000_0532*	outer membrane protein assembly complex subunit YfiO

2.24816	*nusA*	transcription elongation factor NusA
2.24883	*BB2000_3244*	hypothetical protein
2.26151	*rpsT*	30S ribosomal protein S20
2.26366	*BB2000_2949*	dihydrodipicolinate synthase-family protein
2.27994	*fliS*	flagellar protein FliS
2.28019	*BB2000_0653*	hypothetical protein
2.2813	*fkpA*	FKBP-type peptidyl-prolyl cis-trans isomerase
2.29202	*BB2000_3328*	hypothetical protein
2.29483	*BB2000_0021*	hypothetical protein
2.29493	*BB2000_1031*	lipoprotein
2.29551	*trxA*	thioredoxin
2.29561	*BB2000_0725*	probable transporter
2.30025	*BB2000_1041*	hypothetical protein
2.30546	*BB2000_0946*	hypothetical protein
2.31165	*cmk*	cytidylate kinase
2.31834	*ptsI*	phosphoenolpyruvate-protein phosphotransferase
2.32395	*BB2000_1825*	phage protein
2.33486	*BB2000_2653*	chitin binding protein
2.33739	*rpmB*	50S ribosomal protein L28
2.34384	*msrB*	peptide methionine sulfoxide reductase
2.34565	*BB2000_3399*	hypothetical protein

2.35404	*rplA*	50S ribosomal protein L1
2.35487	*rmf*	ribosome modulation factor
2.35971	*lrp*	leucine-responsive transcriptional regulator
2.36114	*ftsK*	cell division protein (DNA translocase)
2.37187	*BB2000_0771*	recombination factor protein RarA
2.38002	*uspE*	universal stress protein UspE
2.38596	*fnr*	fumarate/nitrate reduction transcriptional regulator
2.3922	*rpsR*	30S ribosomal protein S18
2.40355	*BB2000_0573*	hypothetical protein
2.40947	*BB2000_0792*	hypothetical protein
2.41343	*BB2000_1844*	lipoprotein
2.41575	*secG*	protein-export membrane protein
2.43869	*rpsO*	30S ribosomal protein S15
2.44193	*BB2000_1419*	hypothetical protein
2.45316	*BB2000_1829*	phage antitermination protein
2.48477	*BB2000_1828*	phage holin (lysis protein)
2.48477	*sodA*	superoxide dismutase [Mn]
2.48802	*csaA*	protein secretion chaperone
2.49849	*BB2000_1070*	transcriptional regulator
2.50114	*BB2000_1071*	fimbrial subunit
2.50209	*thrS*	threonyl-tRNA synthetase
2.50447	*infC*	translation initiation factor IF-3

2.50743	*rplT*	50S ribosomal protein L20
2.5128	*pheS*	phenylalanyl-tRNA synthetase alpha chain
2.51809	*pheT*	phenylalanyl-tRNA synthetase subunit beta
2.52214	*ihfA*	integration host factor subunit alpha
2.53924	*btuD*	vitamin B12 import ATP-binding protein
2.55988	*arnB*	UDP-4-amino-4-deoxy-L-arabinose--oxoglutarate aminotransferase
2.56167	*arnC*	undecaprenyl phosphate 4-deoxy-4-formamido-L-arabinose transferase
2.56238	*arnA*	bifunctional UDP-glucuronic acid decarboxylase/UDP-4-amino-4-deoxy-L-arabinose formyltransferase
2.56558	*BB2000_1083*	polysaccharide deacetylase
2.57237	*arnT*	4-amino-4-deoxy-L-arabinose transferase
2.58146	*BB2000_1085*	hypothetical protein
2.58165	*BB2000_1086*	hypothetical protein
2.58412	*gltA*	type II citrate synthase
2.58699	*trmD*	tRNA (guanine-N1)-methyltransferase
2.5922	*rpsD*	30S ribosomal protein S4
2.59794	*fliF*	flagellar MS-ring protein
2.6035	*fliH*	flagellar assembly protein H
2.60543	*fliI*	flagellum-specific ATP synthase
2.61728	*BB2000_1824*	phage protein

2.61805	*ompA*	outer membrane protein A
2.62741	*priB*	primosomal replication protein N
2.63659	*dps*	DNA starvation/stationary phase protection protein Dps
2.63831	*metJ*	transcriptional repressor protein MetJ
2.64642	*atpI*	F0F1 ATP synthase subunit I
2.65607	*holD*	DNA polymerase III subunit psi
2.66553	*flgD*	basal-body rod modification protein
2.66643	*fis*	DNA-binding protein Fis
2.66717	*dusB*	tRNA-dihydrouridine synthase B
2.66717	*BB2000_2967*	iron ABC transporter, substrate-binding protein
2.66717	*BB2000_3143*	hypothetical protein
2.66717	*BB2000_2855*	signal sensing protein
2.67152	*nusB*	transcription antitermination protein NusB
2.67459	*flgE*	flagellar hook protein FlgE
2.68283	*BB2000_1204*	hypothetical protein
2.68443	*BB2000_1756*	hypothetical protein
2.68991	*acpP*	acyl carrier protein
2.6928	*fabF*	3-oxoacyl-(acyl carrier protein) synthase II
2.69332	*ppsA*	phosphoenolpyruvate synthase
2.70672	*infA*	translation initiation factor IF-1

2.70948	*aat*	leucyl/phenylalanyl-tRNA--protein transferase
2.71304	*cydC*	cysteine/glutathione ABC transporter membrane/ATP-binding component
2.72113	*cydD*	cysteine/glutathione ABC transporter membrane/ATP-binding component
2.73101	*atpB*	ATP synthase A chain
2.73299	*BB2000_2806*	intracellular sulfur oxidation protein
2.73299	*BB2000_2950*	hypothetical protein
2.73444	*tufB*	elongation factor Tu
2.73554	*BB2000_3500*	acetyltransferase
2.73716	*crp*	cAMP-regulatory protein
2.75141	*ydgA*	hypothetical protein
2.75171	*suhB*	inositol monophosphatase
2.77913	*rpmF*	50S ribosomal protein L32
2.80008	*ribH*	6, 7-dimethyl-8-ribityllumazine synthase
2.82143	*BB2000_2828*	hypothetical protein
2.83558	*atpE*	F0F1 ATP synthase subunit C
2.84894	*crr*	glucose-specific PTS system component
2.85653	*proQ*	putative solute/DNA competence effector
2.86937	*rpsC*	30S ribosomal protein S3
2.87388	*ihfB*	integration host factor subunit beta
2.87807	*ndk*	nucleoside diphosphate kinase

2.88299	*icd*	isocitrate dehydrogenase
2.89118	*lpxC*	UDP-3-O-[3-hydroxymyristoyl] N-acetylglucosamine deacetylase
2.89813	*rplR*	50S ribosomal protein L18
2.90167	*rplY*	50S ribosomal protein L25
2.90167	*BB2000_1795*	hypothetical protein
2.90167	*flgA*	flagella basal body P-ring formation protein
2.90167	*rnpA*	ribonuclease P
2.90167	*rpsH*	30S ribosomal protein S8
2.90167	*fusA*	elongation factor G (EF-G)
2.9107	*rimM*	16S rRNA-processing protein RimM
2.9165	*tolB*	translocation protein TolB
2.95158	*rplN*	50S ribosomal protein L14
2.95832	*budA*	alpha-acetolactate decarboxylase
2.95947	*rpsP*	30S ribosomal protein S16
2.97887	*BB2000_3459*	hypothetical protein
2.98135	*prsA*	ribose-phosphate pyrophosphokinase
2.98876	*rpmD*	50S ribosomal protein L30
3.01302	*rplE*	50S ribosomal protein L5
3.01933	*BB2000_2388*	oxidoreductase
3.01933	*ribB*	3, 4-dihydroxy-2-butanone 4-phosphate synthase
3.03474	*rplP*	50S ribosomal protein L16

3.05733	*rpsQ*	30S ribosomal protein S17
3.06819	*rpsG*	30S ribosomal protein S7
3.07376	*mipA*	MltA-interacting protein precursor
3.07972	*dadB*	alanine racemase, catabolic
3.08307	*dadA*	D-amino acid dehydrogenase small subunit
3.08856	*pal*	peptidoglycan-associated outer membrane lipoprotein
3.09235	*BB2000_1584*	transcriptional regulator
3.09882	*flgB*	flagellar basal body rod protein FlgB
3.11079	*BB2000_0342*	transcriptional regulator
3.11822	*rpsJ*	30S ribosomal protein S10
3.12247	*fliD*	flagellar capping protein
3.14106	*sdhA*	succinate dehydrogenase flavoprotein subunit
3.14414	*sucA*	2-oxoglutarate dehydrogenase E1 component
3.15071	*sucB*	dihydrolipoamide succinyltransferase component of 2-oxoglutarate dehydrogenase complex
3.16157	*sucC*	succinyl-CoA synthetase subunit beta
3.20086	*sucD*	succinyl-CoA synthetase alpha chain
3.20903	*BB2000_0639*	hypothetical protein
3.21287	*BB2000_2819*	methyl-accepting chemotaxis protein
3.21951	*rplV*	50S ribosomal protein L22
3.24112	*BB2000_1483*	hypothetical protein

3.26729	*BB2000_1956*	lipoprotein
3.26729	*BB2000_2387*	hypothetical protein
3.26729	*rplX*	50S ribosomal protein L24
3.31065	*BB2000_0110*	hypothetical protein
3.3325	*BB2000_2655*	hypothetical protein
3.3325	*rplD, rplW, rplB*	50S ribosomal protein L4, 50S ribosomal protein L23, 50S ribosomal protein L2
3.35328	*fliA*	flagellar biosynthesis sigma factor
3.38664	*rplC*	50S ribosomal protein L3
3.39462	*BB2000_1381*	outer membrane protein (attachment invasion locus protein)
3.42274	*emrR*	transcriptional repressor MprA
3.43981	*tufB*	elongation factor Tu
3.46147	*fliE*	flagellar hook-basal body complex protein
3.50226	*rpsN*	30S ribosomal protein S14
3.5091	*rpmH*	50S ribosomal protein L34
3.51141	*fumC*	fumarate hydratase
3.51141	*BB2000_1316*	hypothetical protein
3.51141	*intB*	prophage integrase
3.51141	*flgM, flgN*	anti-sigma28 factor FlgM, flagella synthesis protein
3.51141	*cspA*	cold shock protein
3.51141	*BB2000_1717*	hypothetical protein

3.51141	*fliZ*	flagella biosynthesis protein FliZ
3.51141	*BB2000_1015*	lipase
3.51782	*BB2000_0873*	hypothetical protein
3.5361	*BB2000_0874*	hydrolase
3.54258	*cspB*	cold shock protein
3.56173	*ddg*	cold-induced palmitoleoyl transferase
3.56379	*BB2000_0877*	hypothetical protein
3.59905	*BB2000_0878*	hypothetical protein
3.59909	*BB2000_1586*	hypothetical protein
3.6236	*idrA*	IdrA
3.66151	*BB2000_1097, BB2000_1098, BB2000_1099*	hypothetical protein, fimbrial protein, Fimbrial usher protein
3.70279	*BB2000_1100*	fimbrial chaperone
3.72567	*BB2000_1102*	fimbrial subunit
3.74534	*BB2000_1103*	fimbrial protein
3.75525	*BB2000_1104*	fimbrial protein
3.76036	*BB2000_3499*	lipoprotein
3.79038	*BB2000_0148*	hypothetical protein
3.8123	*BB2000_0579*	hypothetical protein
3.83155	*BB2000_0942*	hypothetical protein
4.02273	*BB2000_2810*	hypothetical protein
4.04203	*BB2000_0184*	hypothetical protein

4.04203	*BB2000_0509*	hypothetical protein
4.10138	*BB2000_1721*	hypothetical protein
4.10384	*BB2000_2492*	hypothetical protein
4.27611	*BB2000_2619*	hypothetical protein
4.31151	*BB2000_2639*	hypothetical protein
4.35192	*BB2000_2663*	hypothetical protein
4.37037	*BB2000_3387*	hypothetical protein
4.37723	*BB2000_0126*	hypothetical protein
4.50389	*BB2000_0881*	hypothetical protein
4.50389	*BB2000_1054*	hypothetical protein
4.50389	*BB2000_1488*	hypothetical protein
4.50389	*BB2000_1610*	hypothetical protein
4.50389	*BB2000_1770*	hypothetical protein
4.50389	*BB2000_2320*	hypothetical protein
4.5988	*BB2000_3091*	hypothetical protein
4.61067	*BB2000_3529*	hypothetical protein
4.64912	*BB2000_1736*	hypothetical protein
4.72832	*BB2000_1947*	hypothetical protein
4.72832	*BB2000_2042*	hypothetical protein
4.72832	*BB2000_2045*	hypothetical protein
4.72832	*arsR*	arsenical resistance operon repressor
4.72832	*BB2000_3033*	hypothetical protein
4.72832	*BB2000_3340*	plasmid-related protein
