# Supplementary material for: Peer pressure from a Proteus mirabilis self-recognition system controls participation in cooperative swarm motility
Source: PLoS Pathog. 2019 Jul 19;15(7):e1007885. doi: 10.1371/journal.ppat.1007885 (PMC6682164; doi:10.1371/journal.ppat.1007885)
Supplement: S5 Table — (PDF) [file ppat.1007885.s014.pdf]

Supplementary Table 5. Annotated (taken from KEGG and COGG databases) functions of the 35 genes for which relative transcript abundance was significantly different in co-swarmed Δ*ids*, clonal CCS02 and clonal CCS06 swarms from a swarm of a clonal wild-type population.

Gene function	Number
Motility and chemotaxis	10
Hypothetical proteins	5
Metabolism	4
Secretion	3
Protein synthesis	3
Other	10
